# Validation of an Instrument for Individuals with Diabetes Mellitus and Hypertension in Primary Health Care

**DOI:** 10.1590/0034-7167-2024-0156

**Published:** 2025-11-03

**Authors:** Carolina Otto, Melissa Orlandi Honório Locks, Luciana Martins da Rosa, Laura Cavalcanti de Farias Brehmer, Vanessa Goulart, Graziani Zanardo

**Affiliations:** I Universidade Federal de Santa Catarina. Florianópolis, Santa Catarina, Brazil

**Keywords:** Diabetes *Mellitus*, Hypertension, Primary Health Care, Nursing, Information Technology, Diabetes *Mellitus*, Hipertensión, Atención Primaria de Salud, Enfermería, Tecnología de la Información

## Abstract

**Objectives:**

to describe the development process and content validity evidence of an instrument designed for use by Community Health Agents during home visits to monitor individuals with Diabetes *Mellitus* and Hypertension in Primary Health Care.

**Methods:**

this methodological study was based on the Double Diamond model and included a literature review, the administration of questionnaires to community health agents and nurses, and content validity assessment by expert nurses. The study was conducted between September 2022 and October 2023 across ten Primary Health Care Units in a municipality in southern Brazil.

**Results:**

a total of 60 professionals participated in the study. The final instrument comprised six domains and achieved a content validity index above 88%, with an overall of 96%.

**Conclusions:**

the proposed instrument demonstrated satisfactory content validity, establishing itself as an accurate and applicable tool for practice, with strong potential for replication in other Primary Health Care contexts.

## RESUMEN

**Objetivos:**

describir el proceso de construcción y la evidencia de validez de contenido de un instrumento destinado a la visita domiciliaria del Agente Comunitario de Salud, en el seguimiento de personas con Diabetes *Mellitus* e Hipertensión Arterial en la Atención Primaria de Salud.

**Métodos:**

estudio metodológico basado en el modelo del Doble Diamante, que incluyó una revisión de la literatura, la aplicación de cuestionarios a agentes comunitarios de salud y enfermeros, además de la validación de contenido por parte de enfermeros expertos. La investigación se llevó a cabo entre septiembre de 2022 y octubre de 2023, en diez Unidades Básicas de Salud de un municipio del sur de Brasil.

**Resultados:**

participaron en el estudio 60 profesionales. El instrumento final se compuso de seis dominios y obtuvo un índice de validez de contenido superior al 88%, con un índice global del 96%.

**Conclusiones:**

el instrumento propuesto presentó un índice de validez de contenido satisfactorio, constituyéndose como una herramienta precisa y aplicable a la práctica, con potencial de replicación en otros contextos de la Atención Primaria de Salud.

## INTRODUCTION

Diabetes Mellitus (DM) and Hypertension (HTN) are two of the most prevalent Non-Communicable Chronic Diseases (NCDs) worldwide, reaching epidemic proportions^(1,2)^. Their progressive increase is associated with social, economic, and technological factors, increased life expectancy, changes in lifestyle habits, and limited access to diagnostic and treatment services^(3)^.

DM is a metabolic disorder characterized by persistent hyperglycemia, which is associated with chronic microvascular and macrovascular complications, increased morbidity, reduced quality of life, and elevated mortality rates^(1,4,5)^. HTN is defined as systolic blood pressure equal to or greater than 140 mmHg and/or diastolic blood pressure (DBP) equal to or greater than 90 mmHg, measured using the correct technique on at least two different occasions, in the absence of antihypertensive medication^(2)^.

Evidence indicates that HTN is one of the main risk factors for cardiovascular diseases (CVD), cerebrovascular diseases, and kidney disorders, which are responsible for high numbers of hospitalizations, outpatient visits, and mortality rates. The main risk factors include age, ethnicity, excess weight, sodium and alcohol intake, physical inactivity, and genetic predisposition^(2,3,6,7)^.

Therefore, Primary Health Care (PHC) plays a central role in health promotion through educational and therapeutic actions that can be developed with patient groups, their families, and the community^(2)^. In this context, nurses play a fundamental role in the development of clinical nursing by using care protocols and educational strategies that go beyond shared knowledge-building, focusing on self-care guidance, identifying and planning new health needs, thereby enabling numerous care strategies^(8,9)^.

Within the PHC setting, home visits are the main activity of Community Health Agents (CHA), who are trained to register and update family records, assist teams in identifying individual and collective risk areas, provide guidance on health promotion and protection, monitor treatment, mobilize the community toward creating health-friendly environments, and report diseases requiring surveillance^(10,11)^.

Furthermore, CHA play a crucial role in the care of individuals with diabetes and HTN, serving as an essential link between the community and health services. Their actions enable the longitudinal follow-up of patients with chronic diseases and their families, facilitating the creation of strong bonds between users and health professionals^(10)^.

In this context, standardizing home visits through a specific instrument is essential for collecting accurate and consistent data; this results in more effective and personalized interventions. Studies show that integrating technologies and support tools into CHA’ activities can significantly improve clinical outcomes for individuals with diabetes and HTN, facilitating early identification of complications and risk factors, and promoting the prevention of adverse outcomes^(11-13)^.

The use of a validated instrument further strengthens the bond between the community and health services, allowing CHA to provide clearer guidance on healthy habits, adherence to medication regimens, and regular monitoring of blood glucose and blood pressure levels^(11,12)^.

Health technologies, therefore, constitute important tools for monitoring DM and HTN. This information can be used to support treatment, chronic disease management, and enhance the capacity and efficiency of health services^(12,13)^. In this way, this instrument can significantly contribute to the workflow of CHA and assist nurses during consultations by gathering data that supports strategic health planning. This strengthens care guidance, health promotion measures, and the prevention of complications, aiming to reduce the impact of DM and HTN through early diagnosis and proper care.

## OBJECTIVES

To describe the development process and content validity evidence of an instrument for home visits conducted by Community Health Agents in the follow-up of individuals with Diabetes Mellitus and Hypertension in Primary Health Care.

## METHODS

### Ethical aspects

The research protocol, originating from a master’s thesis, was reviewed and approved by the Research Ethics Committee of the Federal University of Santa Catarina (UFSC), and the approval document is attached to this submission. Data collection followed the guidelines for research procedures involving any stage conducted in virtual environments, as published by the Brazilian National Research Ethics Commission (CONEP). All participants received the Informed Consent Form (ICF) via email and messaging apps, in PDF format, duly signed by the researchers. By replying to the institutional email containing the ICF and the questionnaire, participants implicitly agreed to the terms of the ICF and consented to participate in the study, thereby dispensing with the need for a signature, while maintaining transparency and traceability in the researcher–participant relationship.

### Study design, setting, and period

This is a methodological study based on the theoretical framework of the “Double Diamond” model^(14)^, which outlines the development and validation of an instrument through four stages: 1) Discover; 2) Define; 3) Develop; and 4) Deliver. The study was conducted between September 2022 and November 2023 across ten PHC Units (UBS in Portuguese) in a municipality in northeastern Santa Catarina, Brazil. This study adheres to the Revised Standards for Quality Improvement Reporting Excellence (SQUIRE 2.0), which provide a framework for reporting new insights into improving health care delivery.

### Population or sample: inclusion and exclusion criteria

The study involved three samples: CHA, nurses, and expert nurses. In the first and second phases of the study, health professionals employed at the institution were invited to participate. The inclusion criteria required participants to be nurses or CHA with at least six months of experience in the Family Health Strategy (FHS), to be fully capable of providing informed consent, and to agree to participate by signing the ICF. Nurses and CHA who were on medical, maternity, or vacation leave were excluded.

At the time of data collection, the municipality had 62 CHA and 16 nurses working in ten PHC Units, organized into 12 FHS teams. Since three of these units had only one registered CHA, the study sought to include at least one CHA from each FHS team to represent the local context of each team. Additionally, to obtain a representative sample of nurses, the goal was to include at least 50% of the practicing nurses.

For the third phase, which involved content validity evidence, the aim was to recruit at least seven experts, ensuring an odd number to avoid ties in the evaluations. Expert nurses were selected through searches on the Lattes Platform. Due to difficulties in receiving responses and the number of experts falling below the recommended minimum, the snowball sampling technique was employed to recruit additional participants^(15)^.

The selection of experts was based on a scoring system adapted from the Fehring model^(16)^, with the following point distribution: holding a master’s or doctoral degree (3 points); having a specialty certification in public health nursing, collective health, or family health (3 points); completion of a residency or specialization in public health, collective health, or family health (3 points); at least two years of current clinical practice in public health, collective health, family health, or care for individuals with DM and HTN (3 points); authorship of research or published articles on the study topic (1 point); abstracts published in the fields of public health, collective health, family health, or care for individuals with DM and HTN (1 point); and participation in courses or conferences in the field with a minimum duration of four hours (1 point). Professionals who achieved a minimum of five points were included in the study. Those who, despite being selected, did not respond after two contact attempts were excluded.

### Study Protocol

In the first stage (discover), a Situational Diagnosis (SD) was carried out with CHA using a semi-structured questionnaire via Google Forms, which included both open- and closed-ended questions. The objective of this SD was to identify the CHA’ roles in care delivery, guidance on care practices, and the follow-up of individuals with DM and HTN, particularly regarding home visits.

In the second stage (define), a narrative literature review was conducted covering the last five years, using the following data sources: Virtual Health Library (BVS), Scientific Electronic Library Online (SciELO), CAPES Journal Portal, Medline via PubMed, as well as technical standards, protocols, and guidelines. The search included the following descriptors: DM, HTN, PHC, Information Technology, and Nursing.

Additionally, a non-systematic bibliographic search was conducted using materials from national health-related organizations focused on diabetes and HTN: the Ministry of Health, the Brazilian Society of Cardiology, and the Brazilian Diabetes Society.

Simultaneously, a semi-structured questionnaire with open- and closed-ended questions was administered to nurses working in the municipality’s health units. The aim was to gather suggestions for content to be included in the instrument and to identify practical issues in the field so that relevant solutions could be incorporated into the tool. The questionnaire was guided by the following core question: “What information about individuals with diabetes and HTN should be included in an instrument to support their follow-up in PHC Care, linked to the CHA home visits?”.

For the first two stages of the study, the invitation to participate included information about the study objectives and access to the Informed Consent Form (ICF). Additionally, a link to the online semi-structured questionnaire, hosted on Google Forms, was sent to participants.

The third stage (develop) focused on designing solutions for the construction of the instrument. The literature review, combined with the analysis of the CHA’ situational diagnosis and the nurses’ questionnaire responses, enabled the identification of key content necessary for the development of the instrument. Accordingly, a prototype of the instrument was structured and organized using Microsoft Word tools, with the relevant content described and categorized.

To ensure the quality and reliability of the instrument’s content, the preliminary version was submitted for expert review. For each response, the Content Validity Index (CVI) was calculated^(17,18)^.

The search for expert nurses was conducted via the Lattes Platform, through the National Council for Scientific and Technological Development (CNPq) portal, using the “curriculum search” function in the “advanced search” option. The following keywords were applied: DM, HTN, PHC, and Nursing (by subject), with filters for academic background and professional experience according to the inclusion criteria. The snowball sampling technique was also used to support recruitment, whereby experts referred other professionals with similar qualifications.

To optimize response time, a single email was sent to each candidate, explaining the purpose of participation and providing links to the ICF (signed by the researchers) and to the electronic evaluation form for the instrument—both created using the Google Forms platform.

In the form, multiple-choice response options were used and set as mandatory, with space provided for open-ended responses. The instrument comprised six domains in total, each of which was rated using a Likert-type scale (1 = inadequate; 2 = partially adequate; 3 = adequate; 4 = fully adequate), along with a field for final comments.

After receiving feedback from the selected experts, modifications were made based on the suggested changes and improvements proposed by the specialists, resulting in the development of the final version of the instrument.

The fourth stage (deliver) involved providing the instrument to the municipality’s PHC Team for future training, digitization, and implementation.

### Data Analysis

The qualitative data obtained from the SD with the CHA and the analysis of the nurses’ questionnaires were compiled and grouped into a single file using Microsoft Word^®^, Office version for Windows 10^®^. The content identified in the literature review and highlighted by participants was grouped by thematic similarity and domain. These were selected up to the point at which data saturation was reached, both in the literature and in the questionnaire analysis, and were analyzed descriptively.

For the qualitative data analysis, the thematic observation method was used^(19)^. This process consisted of three stages: pre-analysis, material exploration, and treatment and interpretation of the results. Information regarding participant characteristics was organized into tables and spreadsheets using Microsoft Excel^®^, Office version for Windows 10^®^, and presented in absolute and relative values, being analyzed descriptively.

Quantitative data, collected from each expert reviewer, were organized into tables using Microsoft Excel^®^, version 2007. The scores assigned to each of the six evaluated domains were reviewed and subsequently analyzed through reflective reading and basic descriptive statistics, presenting both absolute and relative values.

For the content validity analysis, the Content Validity Index (CVI) was calculated. An acceptable agreement rate of 80% (0.8) or higher among expert evaluations was established as the threshold for determining the relevance and/or acceptance of each item, both for individual items and for the overall instrument^(17)^. Scores below 80% (0.8) would have been revised based on the experts’ suggestions and resubmitted for evaluation until the minimum desired reliability of 80% was achieved. However, this was not necessary, as consensus was reached in the first round, with individual CVI scores ranging from 88% to 100%, and an overall CVI of 96%.

## RESULTS

A total of 41 professionals participated in the first and second stages of the study, including 27 CHA and 14 nurses. Regarding gender, 95.1% of participants were women and 4.9% were men. In terms of age, 43.9% of the professionals were between 40 and 50 years old. As for the educational level of the CHA, 55.6% had completed high school. Among the nurses, 50% held a specialization degree in Family Health.

When analyzing the length of service of the professionals in PHC in the municipality, it was found that 31.7% had between six months and two years of experience, 26.8% had been working for over ten years, 22.0% had between two and five years of experience, and 19.5% had between five and ten years of service.

The literature review supported the selection of guiding content for the development of the instrument, such as: comorbidities; lifestyle habits (diet, physical activity, smoking, and drug use); current health status (clinical complaints related to HTN and DM, blood pressure and glucose monitoring, insulin administration care); and health guidance regarding the prevention, management, and avoidance of complications related to HTN and DM.

Based on the data obtained from the SD with the CHA and the analysis of the questionnaire completed by the nurses, this information was organized into five thematic categories, as follows: 1) Difficulties in conducting home visits and monitoring patients; 2) Challenges in treatment adherence; 3) Gaps in care – professional training; 4) Gaps in care – support tools; and 5) Contributions of the CHA to the monitoring of individuals with diabetes and HTN.

Among the difficulties mentioned, gaps were identified in the training of professionals, especially CHA, in relation to managing and addressing the care needs of individuals with diabetes and HTN, which compromises the quality and effectiveness of care. Thus, by highlighting the need for the team to have technological support tools to aid in patient follow-up and care guidance, it was possible to construct and structure an instrument for data collection, recording, and content validity assessment by expert nurses.

For the content validity stage, nine nurses participated. Regarding the professional profile of the expert reviewers, all were women (100%), with the majority aged between 20 and 50 years (66.6%). Of the total, 33.3% held doctoral degrees in Nursing, 88.8% held master’s degrees in Nursing, and 100% had specialist titles. Most of them (88.8%) had more than ten years of professional experience as nurses. Regarding their current employment affiliations, 55.6% worked in Municipal Health Departments and 44.4% in higher education institutions.

The instrument used for content validity, submitted to the expert nurses, consisted of six domains, subdivided into the following aspects:

### Domain 1 – Clinical Diagnosis:

DM; HTN.

### Domain 2 – Identification Data:

Name, preferred name, mother’s name, father’s name, date of birth, sex, relationship status, ethnicity, National Health System (SUS) card number, address, educational level, occupation, phone number, and family caregiver.

### Domain 3 – Comorbidities and Self-Reported Health Conditions:

Type 1 DM and time since diagnosis; Type 2 DM and time since diagnosis; does not know type or time since diagnosis; HTN and time since diagnosis; stroke; high cholesterol; acute myocardial infarction; family history of diabetes; self-reported weight (kg); self-reported height (cm).

### Domain 4 – Lifestyle Habits:

Smoking; drug use; alcohol consumption; physical activity; diet; water intake.

### Domain 5 – Current Health Status:

5.1 – **HTN**: Owns a device for blood pressure (BP) monitoring at home; most recent self-reported BP reading; reported symptoms of high BP.

5.2 – **DM**: Frequency of capillary blood glucose testing; episodes of hypoglycemia and hyperglycemia.

5.3 – **Patient with a DM diagnosis using insulin**: Uses an insulin pen or syringe; storage location of opened and unopened insulin; rotation of injection sites; person responsible for administering insulin; disposal of sharps waste; difficulties related to insulin management.

5.4 – **Investigation of Current Health Status**: Date of most recent blood test; person responsible for picking up medications from the health unit pharmacy; location where medications are stored; whether continuous-use prescriptions are current; presence of lower limb wounds or skin lesions; history of hospitalization related to DM or HTN in the past 30 days; date of most recent appointment at the health unit; vaccination status; and self-care practices.

### Domain 6 – Health Guidance:

Includes guidance to be communicated to the community: education on cardiovascular disease risk factors and preventive recommendations, with emphasis on avoiding harmful habits such as smoking and excessive alcohol consumption; reinforcement of correct medication use as prescribed; assisting patients in following healthcare team recommendations regarding adherence to a healthy, fiber-rich, low-sugar, low-fat diet, with reduced salt and adequate water intake, considering each patient’s reality and needs; encouraging regular physical activity appropriate to the patient’s health condition; reinforcing guidance on proper disposal of sharps waste for patients using insulin therapy—this waste should be discarded in a rigid, puncture-resistant container and delivered to the primary health unit once full; advising and referring patients to schedule appointments at the health unit in the presence of clinical complaints; offering vaccination guidance as recommended by the National Immunization Program and referring patients with overdue vaccinations to the health unit; providing information about the operating hours of the health unit, vaccination room, consultations, exams, and prescription renewals; emphasizing the importance of attending appointments and completing tests requested by the health teams; and encouraging participation in health education groups offered by the health unit.

The main changes suggested by the expert reviewers were: replacing “marital status” with “relationship status”; adding the investigation of gestational HTN; including whether the patient has a glucometer at home and whether it is their own or provided by the health unit; identifying the times of day when capillary blood glucose testing is performed; investigating the most frequent times when hypoglycemia occurs; and removing the investigation of vaccination status.

All of these suggestions were incorporated into the instrument, including the feedback from Judge (J2), who rated Domain 2 as partially adequate and recommended the replacement of “marital status” with “relationship status” (Chart 1). The only recommendation not accepted was the exclusion of the item investigating vaccination status, which is part of Domain 6 (Health Guidance), as this suggestion was made by only one judge (J9).

**Chart 1:** Items comprising the final version of the instrument and CVI calculations, following expert content validation, Florianopolis, Saint Catherine, Brazil, 2023

Items	J1	J2	J3	J4	J5	J6	J7	J8	J9	Individual CVI
**1**	4	3	4	4	3	3	4	3	3	**1.00**
**2**	4	2	3	4	3	3	4	4	3	**0.88**
**3**	4	3	4	4	3	3	4	4	3	**1.00**
**4**	4	4	3	4	3	3	3	4	3	**1.00**
**5**	4	3	4	4	3	3	3	3	3	**1.00**
**6**	4	3	3	4	3	3	4	4	1	**0.88**
**CVI TOTAL**										**0.96**

*J – Judge; CVI – Content Validity Index.*

Thus, it was possible to develop the final version of the instrument following content validation conducted by expert nurses. The instrument was made available to the PHC team of the respective service for implementation within the home visit module used by CHA. The applicability of this instrument will occur during home visits, and through the entry of information into tablets, CHA will be able to apply the instrument and periodically update the data, thereby meeting the needs of the healthcare team.

## DISCUSSION

This study highlights the importance of developing and validating the content of technological tools to support CHA in monitoring individuals with chronic conditions such as HTN and diabetes. Previous experiences, such as the validation of mobile applications for foot care among individuals living with diabetes, have demonstrated that such technologies are not only feasible but also reliable for practical use, promoting self-care and preventing complications such as hospitalizations and amputations^(20)^.

The findings reinforce that technologies developed in the healthcare field must effectively meet identified needs and be relevant to the specific context in which they are applied. This requires researchers to have a deep understanding of the needs and characteristics of the target population^(21)^. Although this aspect was not the central focus of the present study, it is important to emphasize that the relevance of the data collected was strengthened by the active participation of CHA and nurses in the development of the instrument—professionals who are directly involved in patient care. In addition, the incorporation of expert suggestions helped ensure that the proposed technologies address the real needs of the intended context.

During the content validity process, experts recommended the inclusion of a history of gestational HTN. Hypertensive disorders during pregnancy are significant indicators of future cardiovascular risk, as patients who develop any type of HTN during gestation—such as early-onset preeclampsia—are more likely to experience adverse outcomes such as prematurity. These conditions increase the likelihood of developing chronic HTN by three to four times^(2)^.

Experts also suggested that the instrument include an investigation into whether individuals with diabetes use a personal glucometer or one provided by the health unit, in addition to identifying the times of day when capillary blood glucose testing is performed and the most frequent periods during which hypoglycemia occurs. A study conducted at the University Hospital of the Federal District on access to insulin therapy for individuals with diabetes found that the majority of participants obtained their medications—including insulin (93.3%) and oral antidiabetic agents (50.0%)—through public health services, particularly at PHC units^(22)^.

Regarding supplies for capillary blood glucose monitoring—such as glucometers, test strips, and lancets—these were also predominantly obtained through the public health system (93.3%). It is worth noting that the provision of glucometers through the Brazilian Unified Health System (SUS in Portuguese) is regulated by a specific ordinance^(22)^. The expert recommendation to determine whether patients use a personal or health unit-provided glucometer is highly relevant for guiding CHA in the care of individuals with diabetes. These findings underscore the need for effective tools, such as educational technologies for insulin therapy, that address the specific needs of individuals with diabetes in PHC, ensuring personalized care and the prevention of complications.

Regarding glycemic control, one study examined the correlation between nursing consultations and adherence to self-care behaviors and safe insulin therapy practices among individuals with diabetes. It was found that 41.9% of participants reported episodes of hypoglycemia, and 76.7% presented with consistently high blood glucose levels during Self-Monitoring of Blood Glucose (SMBG). With regard to insulin administration, 87.4% of patients self-administered insulin, while 12.6% relied on family members or caregivers^(23)^. These results highlight the importance of continuous follow-up and health education to improve disease management and the quality of life for individuals living with diabetes in PHC settings.

Concerning the item on investigating overdue vaccinations, which is part of the “Health Guidance” domain, only one expert suggested its removal. However, the item was retained. The importance of this monitoring is underscored by the fact that individuals with diabetes and HTN are considered at higher risk for various vaccine-preventable diseases. A study conducted in the United States analyzing hospitalized COVID-19 patients revealed that the most prevalent comorbidities were HTN (56.6%), obesity (41.7%), and diabetes (33.8%)^(24)^.

In this context, validated health technologies play a critical role in supporting effective treatment and the prevention of complications and health deterioration^(25,26)^. Furthermore, such technologies are increasingly present in people’s daily lives and have expanded into the workplace, becoming essential for the development of new tools and educational strategies. Therefore, these technological resources are key allies in public policies aimed at combating diabetes and HTN, while also helping to bridge existing knowledge gaps regarding these conditions.

### Study limitations

One limitation of this study was the difficulty in obtaining completed evaluation instruments from the expert reviewers. Additionally, further research is needed to assess the effectiveness of the instrument’s implementation, including its use in training CHA, analyzing health indicators, and improving treatment adherence—thereby contributing to the prevention of health risks and complications.

### Contributions to Nursing, Health, or Public Policy

The development of technologies within PHC plays a pivotal role in advancing public policies aimed at the prevention of chronic diseases. The creation of instruments aligned with the actual needs of health professionals working in PHC contributes, in the medium term, to the promotion of individualized care. In the long term, these initiatives are expected to significantly reduce the risks of complications related to chronic conditions, thereby improving patients’ quality of life. Furthermore, the model can be adapted and implemented across diverse PHC settings, enhancing its impact and effectiveness. Data collection by CHA provides a detailed view of the local health profile, enabling the healthcare team—particularly nurses—to plan strategic interventions and develop care approaches that are more responsive to community needs.

## CONCLUSIONS

The proposed instrument demonstrated satisfactory content validity indices, establishing itself as a tool capable of standardizing and guiding the work of CHA during home visits with individuals living with DM and HTN in PHC. By validating the content of the data collection instrument, this study represents a significant step forward in enhancing the care provided in PHC, offering professionals effective tools to monitor individuals with diabetes and hypertension more accurately and comprehensively. This integrated approach is essential for promoting health, preventing complications, and ultimately improving the quality of life for people living with these chronic conditions. Additionally, the use of this instrument may contribute to cost reduction and lessen the burden that DM and HTN place on health services.

## Data Availability

The research data are available in a repository: https://doi.org/10.48331/scielodata.X6EGTK.
