# Supplementary material for: Validation of an Instrument for Individuals with Diabetes Mellitus and Hypertension in Primary Health Care
Source: Rev Bras Enferm. 2025 Nov 3;78(4):e20240156. doi: 10.1590/0034-7167-2024-0156 (PMC12584946; doi:10.1590/0034-7167-2024-0156)
Supplement: Supplementary File 1 [file 0034-7167-reben-78-04-e20240156-suppl01.pdf]

Tabela 1-Caracterização dos participantes do estudo do ano de 2023. Florianópolis, SC, Brasil

SEXO	N	%
Feminino	**39**	**95,1**
Masculino	**2**	**4,9**
**IDADE**	**N**	**%**
20 a 30 anos	**10**	**24,4**
30 a 40 anos	**9**	**22,0**
40 a 50 anos	**18**	**43,9**
50 anos ou mais	**4**	**9,8**
**FORMAÇÃO ESCOLARIDADE DOS ACS**	**N**	**%**
Ensino Médio Completo	**15**	**55,6**
Ensino Superior Incompleto	**6**	**22,2**
Ensino Superior Completo	**6**	**22,2**
**ENFERMEIROS ESPECIALIZAÇÃO SAÚDE DA FAMÍLIA**	**N**	**%**
Sim	**7**	**50,0**
Não	**7**	**50,0**
**TEMPO DE ATUAÇÃO NA APS**	**N**	**%**
6 meses a 2 anos	**13**	**31,7**
2 a 5 anos	**9**	**22,0**
5 a 10 anos	**8**	**19,5**
Mais de 10 anos	**11**	**26,8**

Fonte: Elaborado pela pesquisadora (2023).

Organização temática dos questionários dos ACS e enfermeiros:

Dificuldades para visita domiciliar e acompanhamento do paciente

> *Dificuldade em encontrar os pacientes que estão trabalhando em horário comercial* [...]. (ACS3)
>
> *Dificuldade principalmente com os pacientes que trabalham em período integral, pois nos horários de VD não estão na residência para serem orientados.* (E1)
>
> *A maioria desse público está fora do domicílio nos horários de visita das ACS, dificultando o acompanhamento.* (E2)
>
> *Muitos relacionam a falta de acompanhamento com a distância até a unidade e, por isso, acabam não realizando o tratamento devidamente.* (ACS13)

> *Eles, muitas vezes, ficam resistentes ao ir consultar, pois querem apenas renovar receitas e não consultar.* (ACS16)
>
> *Trazer os pacientes para o acompanhamento na unidade e inseri-los nos grupos.* (E3)
>
> *Eles, muitas vezes, ficam resistentes ao ir consultar, pois querem apenas renovar receitas e não consultar.* (ACS4)
>
> [...] *Não há adesão dos pacientes em atividades de grupos.* (ACS5)
>
> *Normalmente, os pacientes de HAS não querem receber os ACS, ou dar informações por medo.* (E11)

Desafios na adesão ao tratamento

> *Dificuldade com o uso correto das medicações e alimentação saudável.* (ACS1)
>
> *Não ter acesso aos medicamentos em uso do paciente, pois, muitas vezes, eles não têm mais a receita ou não querem mostrar, para verificação do uso correto dos medicamentos* [...](E4)
>
> *Muitas vezes, sobre a medicação, falam que estão tomando certinho e vou verificar se estão tomando tudo errado, tomam doses de insulina a mais ou a menos, não têm noção de como aferir uma pressão, mesmo sendo o aparelho de pulso,sempre tem que orientar.* (ACS5)
>
> [...] *a não realização de dietas e alimentação saudável, a não aceitação da doença, por parte dos pacientes* [...] (E5)
>
> *As pessoas idosas são resistentes aos cuidados da alimentação e atividades físicas.* (ACS12)
>
> *No fazer o controle certinho,* [...] *na adesão* [...] *atividade física.* (ACS16)
>
> *Quando o paciente não se importa com a própria saúde e faz maluso das medicações, quando são teimosos, mesmo assim, insisto nas orientações.* (ACS15)
>
> *Dificuldade para seguir o tratamento, devido às questões socioeconômico e à própria cultura.* (ACS26)
>
> *Resistência sobre orientações de dietas.* (ACS14)
>
> *Uma resistência por causa de muitos anos do mal hábito alimentar e de sedentarismo.* (ACS25)

Lacunas no cuidado: formação

Os participantes, inicialmente, ressaltaram as dificuldades existentes para um atendimento de qualidade e mais assertivo, justificadas pela falta de capacitações específicas e continuada para os ACS, sobre o atendimento e as necessidades de cuidados para as pessoas com DM e HA na APS, sendo citadaspor

nove enfermeiras. Também foi mencionada a sobrecarga de trabalho do ACS dentro das unidades de saúde para a execução de funções administrativas, ocasionando numa redução no tempo destinado à realização da visita domiciliar (citado por três enfermeiras), conforme citações:

> *Falta de capacitação técnica para que tenham mais autonomia na assistência aos pacientes.* (E6)
>
> *Sobrecarga com trabalho dentro da UBS, restando pouco tempo para VD de risco e de maior atenção.* (E8)
>
> *Não ser claro na conversa, não saber qual informação passar, não se atentar para situações de alerta.* (E9)
>
> *Profissionais não têm formação para promoção de saúde, ausência de capacidade técnica.* (E14)

Lacunas no cuidado: ferramentas de apoio

> *Planilha Drive de acompanhamento e preenchimento mensal pelos ACS seria excelente.* (E1)
>
> *Talvez uma planilha tipo checklist, onde os ACS possam listar as dificuldades dos pacientes. Dessa maneira, a gente conseguiria traçar um perfil individual, para que as orientações fossem diretamente para cada indivíduo, muitas das informações gerais não condiz com a realidade daquela família ou paciente em questão.* (E2)
>
> *Planilha de verificação individual dos valores pressóricos e glicêmicos. Peso e altura para orientações sobre dieta que poderão ser feitas em consulta para os casos onde há alterações de valores e IMC.* (E7)
>
> *Ter um roteiro do que perguntar, do que observar no Tablet, campos para preencher durante as visitas.* (E13)
>
> *É necessário um levantamento estruturado de dados de pacientes, com e sem adesão ao tratamento, observações das características da microárea, para poder fazer atividades de promoção de saúde mais efetivas, vinculação com família extensiva (parentes, filhos, netos etc), principalmente, no caso de idosos.* (E14)
>
> *Necessita de um instrumento* [...] *destinado ao acompanhamento dos hipertensos e diabéticos, com maiores informações destes pacientes, pois muitos pacientes desconhecem a medicação que utilizam, qual finalidade, não realizam controle adequado de sua pressão arterial, por exemplo.* (E11)
>
> *Relatório contendo se realizam o uso correto dos medicamentos, histórico familiar de doenças cardíacas e outros fatores de risco. Se realizou exames laboratoriais nos últimos 6 meses.* (E4)

Contribuições do agente comunitário de saúde para acompanhamentos das pessoas com diabetes *mellitus* e hipertensão arterial

> *Oriento, quanto ao acompanhamento da condição com exames, cuidados com alimentação e atividades físicas, cuidados com pé diabético. Oriento aferição da PA, minimamente, uma vez ao mês, ingestão correta dos medicamentos, renovação de receitas, exames de sangue semestrais.* (E1)
>
> *Reforçar orientações alimentares de acordo com a realidade do paciente / família; necessidade de acompanhamento médico e enfermeiro na unidade; cuidados com lesões de pele, priorizando lesões em pés, reforçando que o processo de cicatrização é mais lento em tais pacientes.* (E2)
>
> *Questionar a presença de sintomas de elevação e/ou queda do açúcar no sangue aos pacientes com diabetes identificado, e encaminhar para consulta extra* [...]. *Orientar os pacientes sobre a importância da dieta no controle da glicemia, uso correto das medicações, cuidados com os pés, realização de exames laboratoriais e consultas médicas, participação nos grupos, realização de exercícios físicos, diminuição da bebida alcoólica, diminuição do fumo.* (E5)
>
> *Observar condições do ambiente externo e interno do domicílio, verificar se o usuário está utilizando a medicação de forma correta, se existe novas queixas, perguntar sobre novos ferimentos, identificar cuidados com alimentação, verificar carteirinha de vacina. Reforçar a importância da atividade física para os que não têm algumacontraindicação. (*E3)

Tabela 1 - Caracterização dos juízes participantes da pesquisa. Florianópolis, SC, Brasil, 2023

**Sexo**	**N**	**%**
Feminino	**9**	**100,0**
**Idade**	**N**	**%**
20 a 30 anos	**0**	
30 a 40 anos	**3**	**33,3**
40 a 50 anos	**3**	**33,3**
50 anos ou mais	**3**	**33,3**
**Tempo de titulação como Enfermeiro(a)**	**N**	**%**
10 e 20 anos	**4**	**44,4**
20 e 30 anos	**4**	**44,4**
30 anos ou mais	**1**	**11,1**
**Tempo de titulação como Especialista**	**N**	**%**
Menos de 2 anos	**1**	**11,1**
5 e 10 anos	**1**	**11,1**
10 e 20 anos	**3**	**33,3**
20 e 30 anos	**4**	**44,4**
**Tempo de titulação como Mestre**	**N**	**%**
2 e 5 anos	**1**	**11,1**
5 e 10 anos	**3**	**33,3**
10 e 20 anos	**3**	**33,3**
20 e 30 anos	**1**	**11,1**
Não possui mestrado	**1**	**11,1**
**Tempo de titulação como Doutor(a)**	**N**	**%**
5 e 10 anos	**3**	**33,3**
Não possui doutorado	**6**	**66,7**
**Vínculo empregatício atual**		
Secretaria Municipal de Saúde	**5**	**55,6**
Instituição de Ensino Superior	**4**	**44,4**

Fonte: Elaborado pela pesquisadora (2023).
