# Supplementary material for: Validation of an Instrument for Individuals with Diabetes Mellitus and Hypertension in Primary Health Care
Source: Rev Bras Enferm. 2025 Nov 3;78(4):e20240156. doi: 10.1590/0034-7167-2024-0156 (PMC12584946; doi:10.1590/0034-7167-2024-0156)
Supplement: Supplementary File 2 [file 0034-7167-reben-78-04-e20240156-suppl02.pdf]

Instrumento de apoio à visita domiciliar do agente comunitário de saúde para acompanhamento das pessoas com Diabetes *Mellitus* e Hipertensão Arterial

**1. Diagnóstico Clínico**	
☐ Diabetes *Mellitus*<br>☐ Hipertensão Arterial	
**2. Dados de identificação**	
Nome:	Nome social:
Nome da mãe:	Nome do pai:
Data de nascimento:	Situação conjugal:
Sexo:	Escolaridade:
Etnia:	Ocupação:
Cartão SUS:	Telefone:
Endereço:	Responsável familiar:
**3. Comorbidades e condição de saúde referida: Perguntar ao paciente, durante a visita domiciliar, se apresenta**	
☐ Diabetes *Mellitus* tipo 1	Ano do diagnóstico:_____________
☐ Diabetes *Mellitus* tipo 2	Ano do diagnóstico:_____________
☐ Não sabe informar o tipo	Ano do diagnóstico:_____________
☐ Hipertensão arterial	Ano do diagnóstico:_____________
☐ Acidente Vascular Cerebral (derrame cerebral)	☐ Colesterol alto
☐ Infarto Agudo do Miocárdio	☐ Outras
Histórico familiar de diabetes: ☐ sim ☐ não	Quem? ___________________
Peso referido: ___________ kg	Altura referida:___________ cm
**Perguntar apenas às mulheres:**<br>Já teve filhos? ☐ sim ☐ não	**Perguntar apenas às mulheres que já tiveram filhos:**<br>Histórico de diabetes *Mellitus* gestacional:<br>☐ sim ☐ não
**Perguntar apenas às mulheres que já tiveram filhos:**<br>Histórico de hipertensão gestacional:<br>☐ sim ☐ não	

**4. Hábitos de vida:**		
**Tabagismo:**<br>( ) sim ( ) não<br>Quantidade de cigarros por dia:_______	Uso de drogas:<br>( ) sim ( ) não	Tipo:<br>( ) maconha ( ) crack<br>( ) cocaína ( ) outras<br>( ) prefere não responder
**Ingeriu bebida alcoólica no último mês?**<br>( ) sim ( ) não	Tipo de bebida alcoólica:<br>( ) whisky ( ) cachaça ( )vodka<br>( ) cerveja ( ) vinho ( ) outras	Frequência:<br>( ) todos os dias<br>( ) mais de 3 vezes por semana<br>( ) 2 a 3 vezes por semana<br>( ) 1 vez por semana
**Atividade física:**<br>( ) sim ( ) não<br>Tempo da atividade da semana:<br>_______ horas/semanal	Tipo de atividade:<br>( ) caminhada ( ) pedalar<br>( ) musculação ( ) dança<br>( ) outras:_______	Frequência:<br>( ) todos os dias<br>( ) 4 a 5 vezes por semana<br>( ) 2 a 3 vezes por semana<br>( ) 1 vez por semana
**Alimentação:** Quais refeições você faz ao longo do dia?<br>( ) café da manhã<br>( ) lanche da manhã ( ) almoço<br>( ) lanche da tarde ( ) jantar<br>( ) ceia	Consome frutas frescas (não considerar suco de frutas):<br>( ) todos os dias<br>( )às vezes<br>( ) não consome	Consome verduras e/ou legumes (não considerar batata, mandioca/aipim):<br>( ) todos os dias<br>( ) às vezes<br>( ) não consome
Consome hambúrguer e/ou embutidos (presunto, mortadela, salame, linguiça, salsicha):<br>( ) todos os dias<br>( ) às vezes<br>( ) não consome	Consome bebidas adoçadas (refrigerante, suco de caixinha, suco em pó, água de coco de caixinha, suco de fruta com adição de açúcar):<br>( ) todos os dias<br>( ) às vezes<br>( ) não consome	Consome biscoito recheado, doces ou guloseimas (balas, chocolates):<br>( ) todos os dias<br>( ) às vezes<br>( ) não consome

Consome alimentos industrializados (macarrão instantâneo, pipoca de microondas, salgadinhos):<br>( ) todos os dias<br>( ) às vezes<br>( ) não consumo	**Quantidade de água ingerida:**<br>( ) até 5 copos de 200 ml<br>( ) 6 a 8 copos de 200 ml<br>( ) mais de 8 copos de 200ml

5. Situação de saúde atual

5.1 Hipertensão Arterial

Você possui aparelho para aferição da pressão arterial em casa:
( ) sim ( ) não
Com qual frequência você realiza a aferição da pressão arterial:
( ) diariamente ( ) 3 a 4 vezes por semana ( ) 1 vez por semana ( ) bimestral ou semestral ( ) não realizo
Valor da última pressão arterial referida: _____________ mmHg ( ) não lembra

Situações de queixa de sintomas de pressão alta relatada pelo paciente

dor no peito: ( ) sim ( ) não<br>dor na nuca: ( ) sim ( ) não<br>dor de cabeça: ( ) sim ( ) não<br>visão embaçada ou duplicada: ( ) sim ( ) não	tonturas: ( ) sim ( ) não<br>zumbido no ouvido: ( ) sim ( ) não<br>fraqueza: ( ) sim ( ) não<br>palpitações: ( ) sim ( ) não

Frequência da queixa de sintomas de pressão alta:
( ) diariamente ( ) 3 a 4 vezes por semana ( ) 1 vez por semana ( ) mensal ( ) outro período _______

5.1 Diabetes *Mellitus*

Possui glicosímetro em casa: ( ) sim ( ) não
O glicosímetro: ( ) próprio ( ) UBS
Realiza teste de glicemia capilar: ( ) sim ( ) não
Com qual frequência você realiza o teste de glicemia capilar:
( ) diariamente ( ) 3 a 4 vezes por semana ( ) 1 vez por semana ( ) mensal
( ) bimestral ou semestral ( ) não realizo
Qual horário da realização do teste de glicemia capilar:
( ) jejum ( ) antes das refeições ( ) 2 horas após as refeições
Situações de hipoglicemia relatada pelo paciente (**açúcar baixo)**
fome: ( ) sim ( ) não tontura: ( ) sim ( ) não suor frio: ( ) sim ( ) não
tremores ( ) sim ( ) não fadiga, fraqueza e cansaço: ( ) sim ( ) não
visão borrada: ( ) sim ( ) não dor de cabeça: ( ) sim ( ) não
Frequência: ( ) todos os dias ( ) 3 vezes por semana ( ) 1 vez por semana ( ) às vezes
Se presença de hipoglicemia realizar o seguinte questionamento:
Quais horários as hipoglicemias são mais frequentes:
( ) jejum ( ) após o almoço ( ) madrugada ( ) outro __________.
Situações de hiperglicemia relatada pelo paciente (**açúcar alto):**
urina aumentada: ( ) sim ( ) não muita sede: ( ) sim ( ) não
cansaço: ( ) sim ( ) não aumento do apetite: ( ) sim ( ) não
emagrecimento: ( ) sim ( ) não
Frequência: ( ) todos os dias ( ) 3 vezes por semana ( ) 1 vez por semana ( ) às vezes
Apresenta alteração visual: ( ) sim ( ) não

5.3 Se paciente com Diagnóstico de Diabetes *Mellitus* e em uso de insulina, realizar os seguintes questionamentos

Realiza armazenamento da insulina fechada, em sua embalagem, dentro da geladeira, em temperaturas entre 2 e 8 °C: ( ) sim ( ) não
Uso insulina: ( ) caneta ( ) seringa
Realiza armazenamento da insulina aberta (frasco ou caneta descartável ou caneta recarregável, contendo o refil) manter na parte interna inferior da geladeira ou até 30ºC em temperatura ambiente.
( ) sim ( ) não
Realiza rodízio da aplicação da insulina: ( ) sim ( ) não
Responsável pela aplicação da insulina:
( ) paciente ( ) familiar ( ) cuidador ( ) outro:____________
Onde realiza o descarte dos resíduos dos materiais perfuro cortante da aplicação da insulina.
( ) UBS ( ) lixo comum ( ) outros _______________
Paciente, familiar ou cuidador relatou alguma dificuldade ou dúvidas com os cuidados com a insulina:
( ) não ( ) sim Quais:________________

**5.4 Investigação de saúde atual**
Qual foi a última vez que você fez exames de sangue?<br>( ) menos de 6 meses ( ) mais de 6 meses ( ) não lembra<br>Quem é o responsável por retirar o medicamento na farmácia da unidade:<br>( ) paciente ( ) familiar ( ) cuidador ( ) outros __________<br>Em qual local você armazena seus medicamentos?<br>( ) banheiro ( ) cozinha ( ) quarto   ( ) outro:_________<br>Apresenta receita de medicamento de uso contínuo dentro da validade:<br>( ) sim ( ) não<br>Faz uso de medicação conforme orientação médica:<br>( ) sim ( ) não<br>Apresenta ferida (lesão de pele) nos membros inferiores:<br>( ) sim ( ) não<br>Se presença de lesão de pele questionar: está fazendo acompanhamento no serviço de saúde:<br>( ) sim   ( ) não<br>Histórico de hospitalizações relacionadas ao diabetes *Mellitus* ou hipertensão arterial nos últimos 30 dias:<br>( ) sim ( ) não ( ) não soube informar<br>Última consulta da unidade de saúde:<br>( ) menos de 30 dias   (   ) 1 a 2 meses ( ) 3 meses   ( ) 6 meses ( ) 6 a 12 meses   ( ) mais de 12 meses<br>Apresenta alguma vacina em atraso:<br>( ) sim ( ) não /Qual: _____________________<br>Paciente necessita de auxílio para o autocuidado:<br>( ) sim ( ) não<br>Quais: ( ) higiene ( ) alimentação<br>Precisa de ajuda para usar os medicamentos:<br>( ) sim ( ) não<br>Alguém auxilia e confere se os medicamentos estão sendo usados adequadamente:<br>( ) sim ( ) não<br>Capacidade de locomoção:<br>( ) utiliza andador ( ) cadeiras de rodas ( ) acamado ( ) caminhar sem auxílio<br>Paciente apresenta entendimento sobre sua condição de saúde, uso das medicações e das orientações recebidas:<br>( ) sim ( ) não
**6. Orientações de saúde**
(   ) Esclarecer à comunidade sobre os fatores de risco para as doenças cardiovasculares, orientando-a sobre as medidas de prevenção, enfatizando para evitar hábitos prejudiciais, como: tabagismo e consumo excessivo de álcool.<br>(   ) Reforçar sobre o uso correto dos medicamentos, conforme a prescrição médica.<br>(   ) Auxiliar o paciente a seguir as orientações recebidas pela equipe de saúde sobre a adesão de uma dieta saudável e rica em fibras e pobre em açúcares e gorduras, baixo teor de sal e ingestão de água adequada, considerando a realidade e a necessidade de cada paciente.<br>(   ) Ajudar o paciente a seguir as orientações recebidas pela equipe de saúde sobre a prática regular de atividade física apropriada à condição de saúde de cada paciente.<br>(   ) Reforçar as orientações realizadas pela equipe de saúde, que o descarte dos resíduos do paciente em uso de insulinoterapia deverá ser realizado em recipiente rígido resistente e, quando o recipiente estiver cheio, entregar na unidade básica de saúde, para descarte adequado.<br>(   ) Orientar e encaminhar o paciente para o agendamento de consulta na unidade, na presença de queixa clínica.<br>(   ) Realizar orientações sobre a vacinação, conforme preconizado pelo Programa Nacional de Imunização e encaminhar para a unidade de saúde os pacientes com vacinas em atraso.<br>(   ) Orientar sobre horários de funcionamento da unidade, sala de vacinação, consultas, exames e renovação de receitas e reforçar a necessidade do paciente comparecer às consultas e realizar exames solicitados pela equipe de saúde.<br>(   ) Incentivar a participação em grupos de educação em saúde da unidade de saúde.<br>Outras:____
