# Supplementary material for: Validation of an Instrument for Individuals with Diabetes Mellitus and Hypertension in Primary Health Care
Source: Rev Bras Enferm. 2025 Nov 3;78(4):e20240156. doi: 10.1590/0034-7167-2024-0156 (PMC12584946; doi:10.1590/0034-7167-2024-0156)
Supplement: Supplementary File 3 [file 0034-7167-reben-78-04-e20240156-suppl03.pdf]

APÊNDICE D - DIAGNÓSTICO SITUACIONAL COM OS AGENTES COMUNITÁRIOS DE SAÚDE

Diagnóstico situacional

Olá!

Você está sendo convidado a participar de uma pesquisa que faz parte da dissertação de mestrado da Enfa. Carolina Otto, sob orientação da Profª Drª Melissa Orlandi Locks. O objetivo da pesquisa é construir e validar um instrumento informatizado para acompanhamento dos fatores de risco dos pacientes com diagnóstico de Diabetes Mellitus e Hipertensão Arterial para a visita domiciliar do Agente Comunitário na Atenção Primária à Saúde. Este projeto foi avaliado e aprovado pelo Comitê de Ética em Pesquisas com Seres Humanos da Universidade Federal de Santa Catarina (CEPSH-UFSC) seguindo os critérios da Resolução 466/2012 com Certificado de Apresentação de Apreciação Ética (CAAE). Em caso de aceitação clique no link para ter acesso no Termo de Consentimento Livre e Esclarecido(TCLE):

https://drive.google.com/file/d/1HXOUlwVBeddtO8g47hEsaYdUQyXbvqNZ/view?usp=sharing/export?format=pdf

* Indica uma pergunta obrigatória

1. Qual é seu nome? *

____

2. Sexo: *

Marcar apenas uma oval.

( ) Masculino
( ) Feminino
( ) Outro: _______________

3. Qual é sua idade? *

Marcar apenas uma oval.

( ) 20 a 30 anos
( ) 30 a 40 anos
( ) 40 a 50 anos
( ) 50 anos ou mais

4. Qual é sua formação? *

Marcar apenas uma oval.

- Ensino fundamental completo
- Ensino médio incompleto
- Ensino médio completo
- Ensino superior incompleto
- Ensino superior completo

5. Em qual Unidade de Saúde e equipe você atua? *

Marcar apenas uma oval.

- UBS MARIA DE FÁTIMA SILVANO ESF 01
- UBS MARIA DE FÁTIMA SILVANO ESF 02
- UBS JOAQUIM MONTEIRO CABRAL ESF 01
- UBS JOAQUIM MONTEIRO CABRAL ESF 02
- UBS MARIO TITO SALVADOR ESF 01
- UBS MARIO TITO SALVADOR ESF 02
- UBS NICANOR CORRENTE ESF 01
- UBS NICANOR CORRENTE ESF 02
- UBS GENY WESTRUPP KUHNEN ESF 01
- UBS GENY WESTRUPP KUHNEN ESF 02
- UBS MERCEDES BARAUNA
- UBS JOÃO ANTONIO IGNACIO
- UBS ROSALINA MODESTA DA COSTA
- UBS BARRA DO ITAPOCU
- UBS MORRO GRANDE

6. Quanto tempo você atua como Agente Comunitário de Saúde? *

   Marcar apenas uma oval.

   - menos de 6 meses
   - 6 meses a 1 ano
   - 1 a 2 anos
   - 2 a 5 anos
   - 5 a 10 anos
   - mais de 10 anos

7. Qual é seu conhecimento sobre os problemas de saúde dos pacientes hipertensos e diabéticos na sua microárea? *

   Marcar apenas uma oval.

   - não conheço nada
   - conheço muito pouco
   - conheço o suficiente para o meu trabalho
   - conheço bastante

8. Você sabe quais são os principais fatores de risco dos paciente com diagnóstico de Hipertensão Arterial e Diabetes *Mellitus*? *

   Marcar apenas uma oval.

   - não conheço nada
   - conheço muito pouco
   - conheço o suficiente para o meu trabalho
   - conheço bastante

9. Você tem conhecimento sobre o número de pacientes hipertensos e diabéticos * que são tabagistas na sua microárea?

Marcar apenas uma oval.

- não conheço nada
- conheço muito pouco
- conheço o suficiente para o meu trabalho
- conheço bastante

10. Você conhece as complicações que o tabagismo pode causar aos pacientes * com diagnóstico de Hipertensão Arterial e Diabetes *Mellitus*?

Marcar apenas uma oval.

- Sim
- Não

11. Se a resposta a pergunta anterior for sim, liste abaixo as complicações que o tabagismo pode causar aos pacientes com diagnóstico de Hipertensão Arterial e Diabetes *Mellitus:*

____

____

____

____

____

12. Você conhece as complicações que o excesso de peso e a obesidade podem * causar aos pacientes com diagnóstico de Hipertensão Arterial e Diabetes *Mellitus*?

Marcar apenas uma oval.

- Sim
- Não

13. Se a resposta a pergunta anterior for sim, liste abaixo as complicações que o excesso de peso e a obesidade podem causar aos pacientes com diagnóstico de Hipertensão Arterial e Diabetes *Mellitus:*

14. Durante sua visita domiciliar aos pacientes hipertensos e diabéticos você estimula o desenvolvimento de hábitos de vida saudável, tais como: se está cumprindo as orientações de dieta, controle de peso, se reduziu ou parou de fumar? *

Marcar apenas uma oval.

- Sim, em todas as visitas
- Às vezes
- Não realizo esses questionamentos
- Acredito que não seja necessário esses questionamentos

15. Durante sua visita domiciliar aos pacientes hipertensos e diabéticos você orienta a redução do consumo de bebidas alcoólicas ou sua suspensão? *

Marcar apenas uma oval.

- Sim, em todas as vistas
- Às vezes
- Não realizo esses questionamentos
- Acredito que não seja necessário esses questionamentos

16. Durante sua visita domiciliar aos pacientes hipertensos e diabéticos você realiza algum questionamento sobre a importância da realização de atividade físicas? *

Marcar apenas uma oval.

- ◯ Sim, em todas as vistas
- ◯ Às vezes
- ◯ Não realizo esses questionamentos
- ◯ Acredito que não seja necessário esses questionamentos

17. Qual orientações de saúde você realizada durante a visita domiciliar para o seu paciente hipertenso? *

Marque todas que se aplicam.

- ☐ Questiona o comparecimento às consultas na UBS.
- ☐ Questiona a pessoa com hipertensão se está tomando os medicamentos com regularidade.
- ☐ Estimula a participação em grupos de educação em saúde.
- ☐ Questiona se faz acompanhamento da pressão arterial conforme orientação da equipe de saúde.
- ☐ Orienta o agendamento de consulta na UBS quando o paciente queixa-se de agravamento no quadro de saúde.
- ☐ Estimula a participação no grupo de HIPERDIA da UBS.
- ☐ Não faço esses questionamentos.

18. Qual orientações de saúde você realizada durante a visita domiciliar para o seu paciente diabético? *

Marque todas que se aplicam.

- [ ] Questiona o comparecimento às consultas na UBS.
- [ ] Questiona, sempre, à pessoa com diabetes e quando prescritos se ela está tomando com regularidade os medicamentos;
- [ ] Estimula a participação no grupo de HIPERDIA da UBS.
- [ ] Orienta sobre a importância da adesão ao tratamento e seguir as orientações da equipe de saúde
- [ ] Orientar para ter o cuidado corporal redobrado, especialmente com os pés , examinar os pés diariamente e, constatando qualquer alteração, comunicar a
- [ ] Orienta o agendamento de consulta na UBS quando o paciente queixa-se de agravamento no seu quadro de saúde;
- [ ] Estimula a participação em grupos de educação em saúde da UBS;
- [ ] Não faço esses questionamentos.

19. Descreva quais são as principais dificuldades que você encontra durante as visitas domiciliares sobre as orientações de saúde aos pacientes hipertensos e diabéticos. *

____
____
____
____
____

20. Você teria alguma sugestão de instrumentos ou ferramentas que pudesse lhe auxiliar no seu processo de trabalho? *

Marcar apenas uma oval.

- ( ) Instrumento de orientações vinculado ao tablet
- ( ) Protocolo com orientações de cuidados para pacientes hipertensos e diabéticos
- ( ) Roteiro de visita domiciliar
- ( ) Outro: ______________________

21. Você considera importante a desenvolvimento de um instrumento para auxiliar nas visitas domiciliares dos pacientes hipertensos e diabéticos? *

Marcar apenas uma oval.

- Sim
- Não

Este conteúdo não foi criado nem aprovado pelo Google.

Google Formulários

APÊNDICE E - QUESTIONÁRIO COM ENFERMEIROS

Formulário Enfermeiros

Em caso de aceitação clique no link para ter acesso no Termo de Consentimento Livre e Esclarecido(TCLE):

https://drive.google.com/file/d/1uFa_Jzce4BXSad9N9xCvCrODRkEMtrFL/view?usp=drive_link

* Indica uma pergunta obrigatória

1. Qual é seu nome? *

____

2. Qual é sua idade? *

Marcar apenas uma oval.

- ( ) 20 a 30 anos
- ( ) 30 a 40 anos
- ( ) 40 a 50 anos
- ( ) 50 anos ou mais

3. Você possui especialização em Saúde da Família? *

Marcar apenas uma oval.

- ( ) sim
- ( ) não

4. Em qual Unidade de Saúde e equipe você atua? *

Marcar apenas uma oval.

- ( ) UBS MARIA DE FÁTIMA SILVANO ESF 01
- ( ) UBS MARIA DE FÁTIMA SILVANO ESF 02
- ( ) UBS JOAQUIM MONTEIRO CABRAL ESF 01
- ( ) UBS JOAQUIM MONTEIRO CABRAL ESF 02
- ( ) UBS MARIO TITO SALVADOR ESF 01
- ( ) UBS MARIO TITO SALVADOR ESF 02
- ( ) UBS NICANOR CORRENTE ESF 01
- ( ) UBS NICANOR CORRENTE ESF 02
- ( ) UBS GENY WESTRUPP KUHNEN ESF 01
- ( ) UBS GENY WESTRUPP KUHNEN ESF 02
- ( ) UBS MERCEDES BARAUNA
- ( ) UBS JOÃO ANTONIO IGNACIO
- ( ) UBS ROSALINA MODESTA DA COSTA
- ( ) UBS BARRA DO ITAPOCU
- ( ) UBS MORRO GRANDE

5. Qual é seu tempo de atuação na atenção primária no município de Araquari? *

Marcar apenas uma oval.

- ( ) menos de 6 meses
- ( ) 6 meses a 1 ano
- ( ) 1 a 2 anos
- ( ) 2 a 5 anos
- ( ) 5 a 10 anos
- ( ) mais de 10 anos

6. Quais orientações você realiza aos agentes comunitários de saúde para auxiliar na visita domiciliar dos pacientes com diagnóstico de Diabetes Mellitus? *

7. Com qual frequência você realiza essas orientações? *

Marcar apenas uma oval.

- Mensal
- Trimestral
- Semestral
- Anual
- Não estou realizado essas orientações

8. Quais orientações você realiza aos agentes comunitários de saúde para auxiliar na visita domiciliar dos pacientes com diagnóstico de hipertensão arterial? *

9. Com qual frequência você realiza essas orientações? *

Marcar apenas uma oval.

- Mensal
- Trimestral
- Semestral
- Anual
- Não realizado essas orientações

10. Você considera que atualmente os relatórios de informações disponíveis no município sobre os dados dos pacientes com hipertensos e diabéticos na atenção primária são suficientes para definição de perfil epidemiológico? *

Marcar apenas uma oval.

- Sim
- Não

11. Se a resposta a pergunta anterior for não, quais informações você considera necessárias conter nos relatórios? *

____
____
____
____
____

12. Quais as principais dificuldades que você observa que o Agente Comunitário de Saúde tem em relação ao acompanhamento mensal do paciente com diagnóstico de Hipertensão Arterial e Diabetes Mellitus? *

____
____
____
____
____

13. Que tipo de ferramenta de informação você considera necessária que o Agente Comunitário de Saúde verifique durante a visita domiciliar mensal que vão auxiliar sua equipe no planejamento de ações de saúde para os pacientes hipertensos e diabéticos? *

14. Quais informações você considerada importante estar contidas em um instrumento para auxiliar no acompanhamento dos fatores de risco dos pacientes com diagnóstico de Hipertensão Arterial e Diabetes *Mellitus* vinculado com a visita domiciliar do Agente Comunitário de Saúde?" *

Este conteúdo não foi criado nem aprovado pelo Google.

Google Formulários

APÊNDICE F - VALIDAÇÃO DO INSTRUMENTO

VALIDAÇÃO DE CONTEÚDO: Instrumento para acompanhamento dos fatores de risco dos pacientes com diagnóstico de Diabetes *Mellitus* e Hipertensão Arterial pelo Agente Comunitário de Saúde

O presente instrumento faz parte de um projeto do Programa de Pós-Graduação Gestão do Cuidado em Enfermagem – Modalidade Mestrado Profissional da Universidade Federal de Santa Catarina – UFC, que tem por objetivo construir e validar um instrumento informatizado para acompanhamento dos fatores de risco dos pacientes com diagnóstico de Diabetes Mellitus e Hipertensão Arterial Sistêmica vinculado à visita domiciliar do Agente Comunitário na Atenção Primária à Saúde. Esse instrumento foi elaborado a partir das diretrizes da Sociedade Brasileira de Cardiologia, Diabetes Mellitus, Ministério da Saúde, e informações encontradas através da coleta de dados do Diagnóstico Situacional realizado com os Agentes Comunitários de Saúde e entrevista com os enfermeiros que atuam na Atenção Primária à Saúde do município de Araquari/SC no primeiro semestre de 2023. O instrumento é comporto pelos seguintes itens para validação de conteúdo:

1. **Diagnóstico clinico**
2. **Dados de identificação**
3. **Comorbidades e condição de saúde referida**
4. **Hábitos de vida**
5. **Situação de saúde atual**
6. **Orientações de saúde**

Posteriormente, este instrumento será informatizado e disponibilizado para uso do Agentes Comunitários de Saúde do Município de Araquari/SC.

* Indica uma pergunta obrigatória

Informações sobre o preenchimento da avaliação:
Pedimos que leia atentamente cada item, analisando se o **CONTEÚDO** é relevante de ser inserido no instrumento. O instrumento de avaliação utiliza a Escala Likert de 1 a 4 pontos, onde o item 1 representa "Completa Discordância" e o item 4 representa a "Total Concordância", em relação a importância do conteúdo ser incluído na ficha. Solicitamos que caso assinale os itens: "Discordo" ou "Discordo Totalmente", por favor, **FAÇA SEU COMETÁRIO** e sua **SUGESTÃO** para melhorar o item em avaliação. Mesmo não tendo assinalado esses itens, suas atribuições serão de grande importância.
Link para visualizar o instrumento que será validado nas perguntas a seguir:

https://drive.google.com/file/d/1gl_suYI8SHBqbCEkmYF-Ta0BRmCRDfeG/view?usp=sharing/export?format=pdf

Sugestão: Manter o PDF do instrumento aberto enquanto você responde as perguntas para melhorar a compreensão dos itens deste formulário.

1. Nome completo *

____

2. Endereço de email *

____

3. Sexo *

Marcar apenas uma oval.

- ◯ Feminino
- ◯ Masculino
- ◯ Outros

4. Qual é sua idade? *

Marcar apenas uma oval.

- 20 a 30 anos
- 30 a 40 anos
- 40 a 50 anos
- 50 a 60 anos
- 60 anos ou mais

5. Instituição de vínculo *

____

6. Tempo de titulação como Enfermeiro(a): *

Marcar apenas uma oval.

- Menos de 2 anos
- Entre 2 e 5 anos
- Entre 5 e 10 anos
- Entre 10 e 20 anos
- Entre 20 e 30 anos
- 30 anos ou mais

7. Tempo de titulação como Especialista *

Marcar apenas uma oval.

- Menos de 2 anos
- Entre 2 e 5 anos
- Entre 5 e 10 anos
- Entre 10 e 20 anos
- Entre 20 e 30 anos
- 30 anos ou mais

8. Tempo de titulação como Mestre *

Marcar apenas uma oval.

- Menos de 2 anos
- Entre 2 e 5 anos
- Entre 5 e 10 anos
- Entre 10 e 20 anos
- Entre 20 e 30 anos
- 30 anos ou mais
- Não tenho mestrado

9. Tempo de titulação como Doutor(a) *

Marcar apenas uma oval.

- Menos de 2 anos
- Entre 2 e 5 anos
- Entre 5 e 10 anos
- Entre 10 e 20 anos
- Entre 20 e 30 anos
- 30 anos ou mais
- Não tenho doutorado

Analise quanto ao conteúdo se os itens mencionados nos **Dados de Identificação** são **RELEVANTES** quanto a investigação durante a visita domiciliar do Agente Comunitário de Saúde para acompanhamento dos fatores de risco dos pacientes com diagnóstico de Diabetes *Mellitus* e Hipertensão Arterial:

- Nome
- Nome Social
- Nome da mãe
- Nome do pai
- Data de nascimento
- Estado Civil
- Sexo
- Escolaridade
- Etnia
- Ocupação
- Cartão SUS
- Telefone
- Responsável familiar

10. Em relação ao conteúdo relacionado aos **Dados de Identificação:** *

Marcar apenas uma oval.

- ( ) Concordo Totalmente
- ( ) Concordo
- ( ) Discordo
- ( ) DiscordoTotalmente

11. Observações e sugestões *

____

Analise quanto ao conteúdo se os itens mencionados nos dados relacionados as **Comorbidades e Condição de Saúde Referidas** são **RELEVANTES** quanto a investigação durante a visita domiciliar do Agente Comunitário de Saúde para acompanhamento dos fatores de risco dos pacientes com diagnóstico de Diabetes *Mellitus* e Hipertensão Arterial:

- Diabetes mellitus tipo 1 / Tempo que possui diagnóstico:
- Diabetes mellitus tipo 2 / Tempo que possui diagnóstico:
- Não sabe informar o tipo / Tempo que possui diagnóstico:
- Hipertensão arterial / Tempo que possui diagnóstico:
- Acidente Vascular Cerebral (derrame cerebral)
- Colesterol alto
- Infarto Agudo do Miocárdio
- Outras
- Histórico familiar de diabetes: ( ) sim ( ) não Quem?:
- Peso referido: kg
- Altura referida: cm

- **Perguntar apenas às mulheres:**
- Histórico de diabetes mellitus gestacional : ( ) sim ( ) não

12. Em relação ao conteúdo relacionado as **Comorbidades e Condição de Saúde Referida:** *

Marcar apenas uma oval.

- ( ) Concordo Totalmente
- ( ) Concordo
- ( ) Discordo
- ( ) DiscordoTotalmente

13. Observações e sugestões *

___

Analise quanto ao conteúdo se os itens mencionados nos dados **Hábitos de vida** são **RELEVANTES** quanto a investigação durante a visita domiciliar do Agente Comunitário de Saúde para acompanhamento dos fatores de risco dos pacientes com diagnóstico de Diabetes *Mellitus* e Hipertensão Arterial:

- Tabagismo: ( ) sim ( ) Não
- Quantidade de cigarros por dia:______
- Uso de drogas: ( ) sim ( ) não
- Tipo: ( ) Maconha ( ) crack ( ) cocaína ( ) outras ( ) prefere não responder
- Bebidas alcoólicas: ( ) sim ( ) não
- Tipo de bebida alcoólica: ( ) whisky ( ) cachaça ( ) vodka ( ) cerveja ( ) vinho ( ) outras
- Frequencia: ( ) todos os dias ( ) mais de 3 x/semana ( ) 2 -3x/semana ( ) 1x/semana
- Atividade física: ( ) sim ( ) não Tipo de atividade: ( ) caminhada ( ) pedalar ( ) musculação ( ) dança ( ) outras:______
- Frequência: ( ) todos os dias ( ) 4 a 5 vezes por semana ( ) 2 a 3 vezes por semana ( ) 1 vez por semana
- Alimentação: Quais refeições você faz ao longo do dia? ( ) Café da manhã ( ) Lanche da manhã ( ) Almoço ( ) Lanche da tarde ( ) Jantar ( ) Ceia
- Consome frutas frescas (não considerar suco de frutas): ( ) todos os dias ( ) as vezes ( ) não consome
- Consome verduras e/ou legumes (não considerar batata, mandioca/aipim): ( ) todos os dias ( ) as vezes ( ) não consome
- Consome hambúrguer e/ou embutidos (presunto, mortadela, salame, linguiça, salsicha): ( ) todos os dias ( ) as vezes ( ) não consome
- Consome bebidas adoçadas (refrigerante, suco de caixinha, suco em pó, água de coco de caixinha, suco de fruta com adição de açúcar): ( ) todos os dias ( ) as vezes ( ) não consome
- Consome biscoito recheado, doces ou guloseimas (balas, chocolates): ( ) todos os dias ( ) as vezes ( ) não consome
- Consome alimentos industrializados (macarrão instantâneo, pipoca de microondas, salgadinhos): ( ) todos os dias ( ) as vezes ( ) não consumo
- Quantidade de água ingerida: ( ) até 5 copos de 200 ml ( ) 6 a 8 copos de 200 ml ( ) mais de 8 copos de 200m

14. Em relação ao conteúdo relacionado aos **Hábitos de vida:** *

Marcar apenas uma oval.

- Concordo Totalmente
- Concordo
- Discordo
- DiscordoTotalmente

15. Observações e sugestões *

____

Analise quanto ao conteúdo se os itens mencionados nas **Situação de Saúde Atual** são **RELEVANTES** quanto a investigação durante a visita domiciliar do Agente Comunitário de Saúde para acompanhamento dos fatores de risco dos pacientes com diagnóstico de Hipertensão Arterial e Diabetes *Mellitus:*

- **Hipertensão Arterial**
- Você possui aparelho para aferição da PA em casa: ( ) sim ( ) não
- Com qual frequência você realiza a aferição da Pressão Arterial (PA):
  ( ) diariamente ( ) 3 a 4 vezes por semana ( ) 1 vez por semana ( ) não realizo
- Valor da última PA referida: _________ mmHg ( ) não lembra
- Situações de queixa de sintomas de pressão alta relatada pelo paciente (dor no peito, dor na nuca, dor de cabeça, tonturas, zumbido no ouvido, fraqueza, palpitações, visão embaçada ou duplicada):
- ( ) sim ( ) não
- Frequência: ( ) todos os dias ( ) 3 vezes por semana ( ) 1 vez por semana ( ) as vezes
- **Diabetes Mellitus**
- Com qual frequência você realiza o teste de glicemia capilar:

  ( )diariamente ( ) 3 a 4 vezes por semana
- ( ) 1 vez por semana
  ( ) não realizo
- Situações de hipoglicemia relatada pelo paciente (açúcar baixo: fome, tontura, suor frio, tremores, fadiga, fraqueza e cansaço, visão borrada, dor de cabeça): ( ) sim ( ) não
- Frequência: ( ) todos os dias ( ) 3 vezes por semana ( ) 1 vez por semana ( ) as vezes
- Situações de hiperglicemia relatada pelo paciente (açúcar alto: urina aumentada, muita sede, cansaço, aumento do apetite emagrecimento):
  ( ) sim ( ) não
- Frequência: ( ) todos os dias ( ) 3 vezes por semana ( ) 1 vez por semana ( ) as vezes
- Qual foi a última vez que você fez exames de sangue? ( ) menos de 6 meses ( ) mais de 6 meses ( ) não lembra
- Quem é o responsável por retirar o medicamento na farmácia da unidade: ( ) paciente ( ) familiar ( ) cuidador ( ) outros_________
- Em qual local você armazena seus medicamentos? ( ) banheiro ( ) cozinha ( ) quarto ( ) outro:________
  Apresenta receita de medicamento de uso contínuo dentro da validade: ( ) sim ( ) não
- Faz uso de medicação conforme orientação médica: ( ) sim ( ) não
- Apresenta ferida (lesão de pele) nos membros inferiores: ( ) não ( ) sim, está realizando acompanhamento no serviço de saúde: ( ) sim ( ) não
- Histórico de hospitalizações relacionadas ao diabetes mellitus ou hipertensão arterial nos últimos 30 dias: ( ) sim ( ) não ( ) não soube informar
- Última consulta da unidade de saúde: ( ) menos de 30 dias ( ) 1 a 2 meses ( ) 3 meses ( ) 6 meses ( ) 6 a 12 meses ( ) mais de 12 meses
- Apresenta alguma vacina em atraso: ( ) sim ( ) não qual: __________________
- Paciente consegue realizar seu autocuidado relacionado a higiene, alimentação: ( ) sim ( ) não, paciente necessita de auxílio da família ou do cuidador para auxiliar nos cuidados.

- Paciente apresenta entendimento sobre sua condição de saúde, uso das medicações e das orientações recebidas: ( ) sim ( ) não

16. Em relação ao conteúdo relacionados as **Situação de Saúde Atual:** *

Marcar apenas uma oval.

( ) Concordo Totalmente

( ) Concordo

( ) Discordo

( ) DiscordoTotalmente

17. Observações e sugestões *

____

Analise quanto ao conteúdo se os itens mencionados **""Se paciente com Diagnóstico de Diabetes Mellitus e em uso de uso de insulina, realizar os seguintes questionamentos""** são **RELEVANTES** quanto a investigação durante a visita domiciliar do Agente Comunitário de Saúde para acompanhamento dos fatores de risco dos pacientes com diagnóstico de Diabetes *Mellitus:*

- Uso de caneta para insulina: ( ) sim ( ) não
- Uso de seringa para insulina: ( ) sim ( ) não
- Realiza armazenamento da insulina aberta: ( ) fora da geladeira ( ) porta da geladeira ( ) prateleiras da geladeira
- Realiza armazenamento da insulina fechada: ( ) fora da geladeira ( ) porta da geladeira ( ) prateleiras da geladeira
- Realiza rodízio da aplicação da insulina: ( ) sim ( ) não
- Responsável pela aplicação da insulina: ( ) paciente ( ) familiar ( ) cuidador ( ) outro:___________
- Onde descarta os resíduos dos materiais perfuro cortante da aplicação da insulina. ( ) UBS ( ) lixo comum ( ) outros_______________
- Paciente, familiar ou cuidador relatou alguma dificuldade ou dúvidas com os cuidados com a insulina: ( ) não ( ) sim quais:________________

18. Em relação ao conteúdo relacionado "**Se paciente com Diagnóstico de Diabetes Mellitus e em uso de uso de insulina, realizar os seguintes questionamentos:"** *

Marcar apenas uma oval.

- Concordo Totalmente
- Concordo
- Discordo
- DiscordoTotalmente

19. Observações e sugestões *

20. Observações e sugestões *

Analise quanto ao conteúdo se os itens mencionados nos dados **Orientações de Saúde** são **RELEVANTE**S quanto a orientação durante a visita domiciliar do Agente Comunitário de Saúde para acompanhamento dos fatores de risco dos pacientes com diagnóstico de Diabetes *Mellitus e Hipertensão Arterial:*

( ) Esclarecer a comunidade sobre os fatores de risco para as doenças cardiovasculares, orientando-a sobre as medidas de prevenção, enfatizando para evitar hábitos prejudiciais, como tabagismo e consumo excessivo de álcool.

( ) Reforçar sobre o uso correto dos medicamentos conforme a prescrição médica.

( ) Auxiliar o paciente a seguir as orientações recebidas pela equipe de saúde sobre a adesão de uma dieta saudável e rica em fibras e pobre em açúcares e gorduras, baixo teor de sal e ingestão de água adequada, considerando a realidade e a necessidade de cada paciente.

( ) Ajudar o paciente a seguir as orientações recebidas pela equipe de saúde sobre a prática regular de atividade física apropriada para a condição de saúde de cada paciente.

( ) Reforçar as orientações realizadas pela equipe de saúde que o descarte dos resíduos do paciente em uso de insulinoterapia deverá ser realizado em recipiente rígido resistente e quando o recipiente estiver cheio, entregar na unidade básica de saúde para descarte adequado.

( ) Orientar e encaminhar o paciente para o agendamento de consulta na unidade, na presença de queixa clínica.

( ) Realizar orientações sobre o vacinação conforme preconizado pelo Programa Nacional de Imunização e encaminhar para a unidade de saúde os pacientes com vacinas em atraso.

( ) Orientar sobre horários de funcionamento da unidade, sala de vacinação, consultas, exames e renovação de receitas e reforçar a necessidade do paciente comparecer às consultas e realizar exame solicitados pelas equipe de saúde.

( ) Incentivar a participação em grupos de educação em saúde da unidade de saúde.

Outras:____
____

21. Em relação ao conteúdo relacionado as **Orientações de saúde:** *

Marcar apenas uma oval.

- ⬭ Concordo Totalmente
- ⬭ Concordo
- ⬭ Discordo
- ⬭ DiscordoTotalmente

22. Observações e sugestões *

____

23. Você sugere mais alguma informação que não foram mencionada neste instrumento? *

Este conteúdo não foi criado nem aprovado pelo Google.

Google Formulários

APÊNDICE H – INSTRUMENTO DE VALIDAÇÃO

Instrumento para acompanhamento dos fatores de risco dos pacientes com diagnóstico de Diabetes *Mellitus* e Hipertensão Arterial pelo Agente Comunitário de Saúde

**1. Diagnóstico Clinico:**	
☐ Diabetes *Mellitus*<br>☐ Hipertensão Arterial	
**2. Dados de identificação:**	
Nome:	Nome social:
Nome da mãe:	Nome do pai
Data de nascimento:	Estado Civil:
Sexo:	Escolaridade:
Etnia:	Ocupação:
Cartão SUS:	Telefone:
Endereço:	Responsável familiar:
**3. Comorbidades e condição de saúde referida: Perguntar ao paciente durante a visita domiciliar se apresenta:**	
☐ Diabetes *mellitus* tipo 1	Tempo que possui diagnóstico:_____________
☐ Diabetes *mellitus* tipo 2	Tempo que possui diagnóstico:_____________
☐ Não sabe informar o tipo	Tempo que possui diagnóstico:_____________
☐ Hipertensão arterial	Tempo que possui diagnóstico:_____________
☐ Acidente Vascular Cerebral (derrame cerebral)	☐ Colesterol alto
☐ Infarto Agudo do Miocárdio	☐ Outras
Histórico familiar de diabetes: ☐ sim ☐ não Quem?: ____________________	
Peso referido: ___________kg	Altura referida:___________ cm
**Perguntar apenas às mulheres:**<br>Histórico de diabetes *mellitus* gestacional : ☐ sim ☐ não	

**4. Hábitos de vida:**		
**Tabagismo:** ( ) sim ( ) Não<br>Quantidade de cigarros por dia:_______	Uso de drogas: ( ) sim ( ) não	Tipo: ( ) Maconha ( ) crack ( ) cocaína<br>( ) outras ( ) prefere não responder
**Bebidas alcoólicas:** ( ) sim ( ) não	Tipo de bebida alcoólica: ( ) whisky ( ) cachaça ( ) vodka ( ) cerveja ( ) vinho ( ) outras	Frequência: ( ) todos os dias ( ) mais de 3 x/semana ( ) 2 -3x/semana ( ) 1x/semana
**Atividade física:** ( ) sim ( ) não	Tipo de atividade: ( ) caminhada<br>( ) pedalar ( ) musculação ( ) dança<br>( ) outras:_______	Frequência: ( ) todos os dias ( ) 4 a 5 vezes por semana ( ) 2 a 3 vezes por semana<br>( ) 1 vez por semana
**Alimentação:** Quais refeições você faz ao longo do dia? ( ) Café da manhã ( ) Lanche da manhã ( ) Almoço ( ) Lanche da tarde<br>( ) Jantar ( ) Ceia	Consome frutas frescas (não considerar suco de frutas): ( ) todos os dias ( ) as vezes ( ) não consome	Consome verduras e/ou legumes (não considerar batata, mandioca/aipim): ( ) todos os dias ( ) as vezes ( ) não consome
Consome hambúrguer e/ou embutidos (presunto, mortadela, salame, linguiça, salsicha): ( ) todos os dias ( ) as vezes ( ) não consome	Consome bebidas adoçadas (refrigerante, suco de caixinha, suco em pó, água de coco de caixinha, suco de fruta com adição de açúcar):<br>( ) todos os dias ( ) as vezes ( ) não consome	Consome biscoito recheado, doces ou guloseimas (balas, chocolates): ( ) todos os dias ( ) as vezes ( ) não consome
Consome alimentos industrializados (macarrão instantâneo, pipoca de microondas, salgadinhos): ( ) todos os dias ( ) as vezes ( ) não consumo	**Quantidade de água ingerida:**<br>( ) até 5 copos de 200 ml ( ) 6 a 8 copos de 200 ml ( ) mais de 8 copos de 200ml	

**5. Situação de saúde atual:**	
**Hipertensão Arterial**	**Diabetes Mellitus**
Você possui aparelho para aferição da PA em casa: ( ) sim ( ) não	Com qual freqüência você realiza o teste de glicemia capilar:

Com qual freqüência você realiza a aferição da Pressão Arterial (PA):<br>( ) diariamente ( ) 3 a 4 vezes por semana ( ) 1 vez por semana ( ) não realizo<br>**Valor da última PA referida: ____________ mmHg ( ) não lembra**<br>Situações de queixa de sintomas de **pressão alta** relatada pelo paciente (dor no peito, dor na nuca, dor de cabeça, tonturas, zumbido no ouvido, fraqueza, palpitações, visão embaçada ou duplicada).<br>( ) sim ( ) não<br>Frequência: ( ) todos os dias ( ) 3 vezes por semana ( ) 1 vez por semana ( ) as vezes	( )diariamente ( ) 3 a 4 vezes por semana ( ) 1 vez por semana ( ) não realizo<br>Situações de hipoglicemia relatada pelo paciente (**açúcar baixo**: fome, tontura, suor frio, tremores, fadiga, fraqueza e cansaço, visão borrada, dor de cabeça): ( ) sim ( ) não<br>Frequência: ( ) todos os dias ( ) 3 vezes por semana ( ) 1 vez por semana ( ) as vezes<br>Situações de hiperglicemia relatada pelo paciente (**açúcar alto:** urina aumentada, muita sede, cansaço, aumento do apetite emagrecimento):<br>( ) sim ( ) não<br>Frequência: ( ) todos os dias ( ) 3 vezes por semana ( ) 1 vez por semana ( ) as vezes

Qual foi a última vez que você fez exames de sangue? ( ) menos de 6 meses ( ) mais de 6 meses ( ) não lembra

Quem é o responsável por retirar o medicamento na farmácia da unidade: ( ) paciente ( ) familiar ( ) cuidador ( ) outros__________

Em qual local você armazena seus medicamentos? ( ) banheiro ( ) cozinha ( ) quarto ( ) outro:_________

Apresenta receita de medicamento de uso contínuo dentro da validade: ( ) sim ( ) não

Faz uso de medicação conforme orientação médica: ( ) sim ( ) não

Apresenta ferida (lesão de pele) nos membros inferiores: ( ) não ( ) sim, está fazendo acompanhamento no serviço de saúde: ( ) sim ( ) não

Histórico de hospitalizações relacionadas ao diabetes *mellitus* ou hipertensão arterial nos últimos 30 dias: ( ) sim ( ) não ( ) não soube informar

Última consulta da unidade de saúde: ( ) menos de 30 dias ( ) 1 a 2 meses ( ) 3 meses ( ) 6 meses ( ) 6 a 12 meses ( ) mais de 12 meses

Apresenta alguma vacina em atraso: ( ) sim ( ) não qual: _____________________

Paciente consegue realizar seu autocuidado relacionado a higiene, alimentação: ( ) sim ( ) não, paciente necessita de auxílio da família ou do cuidador para auxiliar nos cuidados.

Paciente apresenta entendimento sobre sua condição de saúde, uso das medicações e das orientações recebidas: ( ) sim ( ) não

Se paciente com Diagnóstico de Diabetes *Mellitus* e em uso de uso de insulina, realizar os seguintes questionamentos:

Uso de caneta para insulina: ( ) sim ( ) não

Uso de seringa para insulina: ( ) sim ( ) não

Realiza armazenamento da insulina aberta: ( ) fora da geladeira ( ) porta da geladeira ( ) prateleiras da geladeira

Realiza armazenamento da insulina fechada: ( ) fora da geladeira ( ) porta da geladeira ( ) prateleiras da geladeira

Realiza rodízio da aplicação da insulina: ( ) sim ( ) não

Responsável pela aplicação da insulina: ( ) paciente ( ) familiar ( ) cuidador ( ) outro:____________

Onde descarta os resíduos dos materiais perfuro cortante da aplicação da insulina. ( ) UBS ( ) lixo comum ( ) outros________________

Paciente, familiar ou cuidador relatou alguma dificuldade ou dúvidas com os cuidados com a insulina: ( ) não ( ) sim quais:_________________

6. Orientações de saúde:

( ) Esclarecer a comunidade sobre os fatores de risco para as doenças cardiovasculares, orientando-a sobre as medidas de prevenção, enfatizando para evitar hábitos prejudiciais, como tabagismo e consumo excessivo de álcool.

( ) Reforçar sobre o uso correto dos medicamentos conforme a prescrição médica.

( ) Auxiliar o paciente a seguir as orientações recebidas pela equipe de saúde sobre a adesão de uma dieta saudável e rica em fibras e pobre em açúcares e gorduras, baixo teor de sal e ingestão de água adequada, considerando a realidade e a necessidade de cada paciente.

( ) Ajudar o paciente a seguir as orientações recebidas pela equipe de saúde sobre a prática regular de atividade física apropriada para a condição de saúde de cada paciente.

( ) Reforçar as orientações realizadas pela equipe de saúde que o descarte dos resíduos do paciente em uso de insulinoterapia deverá ser realizado em recipiente rígido resistente e quando o recipiente estiver cheio, entregar na unidade básica de saúde para descarte adequado.

( ) Orientar e encaminhar o paciente para o agendamento de consulta na unidade, na presença de queixa clínica.

( ) Realizar orientações sobre o vacinação conforme preconizado pelo Programa Nacional de Imunização e encaminhar para a unidade de saúde os pacientes com vacinas em atraso.

( ) Orientar sobre horários de funcionamento da unidade, sala de vacinação, consultas, exames e renovação de receitas e reforçar a necessidade do paciente comparecer às consultas e realizar exame solicitados pelas equipe de saúde.

( ) Incentivar a participação em grupos de educação em saúde da unidade de saúde.

Outras:____
