# Supplementary material for: Validation of an Instrument for Individuals with Diabetes Mellitus and Hypertension in Primary Health Care
Source: Rev Bras Enferm. 2025 Nov 3;78(4):e20240156. doi: 10.1590/0034-7167-2024-0156 (PMC12584946; doi:10.1590/0034-7167-2024-0156)
Supplement: Translation

# Translation: Validação de instrumento para pessoas com *Diabetes Mellitus* e Hipertensão Arterial na Atenção Primária

**DOI:** 10.1590/0034-7167-2024-0156pt

**Authors:** Carolina Otto, Melissa Orlandi Honório Locks, Luciana Martins da Rosa, Laura Cavalcanti de Farias Brehmer, Vanessa Goulart, Graziani Zanardo

**Affiliations:** I Universidade Federal de Santa Catarina. Florianópolis, Santa Catarina, Brasil

**Keywords:** Diabetes *Mellitus*, Hipertensão Arterial, Atenção Primária à Saúde, Enfermagem, Tecnologia de Informação

## Abstract

**Objetivos:**

descrever o processo de construção e evidência de validade de conteúdo de um instrumento para a visita domiciliar do Agente Comunitário de Saúde, no acompanhamento de pessoas com Diabetes *Mellitus* e Hipertensão Arterial, na Atenção Primária à Saúde.

**Métodos:**

estudo metodológico baseado no modelo do Duplo Diamante, que incluiu revisão da literatura, aplicação de questionário com agentes comunitários de saúde e enfermeiros, além da evidência de validade de conteúdo com enfermeiros *experts*. A pesquisa foi realizada entre setembro de 2022 e outubro de 2023, em dez Unidades Básicas de Saúde de um município do Sul do Brasil.

**Resultados:**

participaram do estudo 60 profissionais. O instrumento final foi composto por seis domínios e obteve índice de validade de conteúdo superior a 88%, com índice global de 96%.

**Conclusões:**

o instrumento proposto apresentou índice de validade de conteúdo satisfatório, configurando-se como uma ferramenta acurada e aplicável à prática, com potencial de replicação em outras realidades da Atenção Primária à Saúde.

## INTRODUÇÃO

O Diabetes *Mellitus* (DM) e a Hipertensão Arterial (HA) são duas das Doenças Crônicas Não Transmissíveis (DCNT) mais prevalentes no mundo, atingindo proporções epidêmicas^(1,2)^. O seu aumento progressivo está relacionado a fatores sociais, econômicos e tecnológicos, aumento da expectativa de vida, mudança de hábitos de vida, além da falta de acesso a serviços de diagnóstico e tratamento^(3)^.

O DM é um distúrbio metabólico caracterizado por hiperglicemia persistente, que está associado a complicações crônicas micro e macrovasculares, aumento da morbidade, redução da qualidade de vida e elevação da taxa de mortalidade^(1,4,5)^. A HA é definida pela alteração dos níveis da Pressão Arterial Sistólica (PAS) maior ou igual a 140 mmHg e/ou Pressão Arterial Diastólica (PAD) maior ou igual a 90 mmHg, medida com a técnica correta, em pelo menos duas ocasiões diferentes, na ausência de medicação anti-hipertensiva^(2)^.

Evidências apontam que a HA é um dos principais fatores de risco para doenças cardiovasculares (DCV), doenças cerebrovasculares e renais, responsáveis por elevados números de hospitalizações, atendimentos ambulatoriais e índices de óbitos. Seus principais fatores de risco incluem: idade, etnia, excesso de peso, ingestão de sódio, ingestão de álcool, sedentarismo e fatores genéticos^(2,3,6,7)^.

Dessa forma, é na Atenção Primária à Saúde (APS) que se desenvolve a promoção da saúde, por meio da realização de ações educativas e terapêuticas, que podem ser elaboradas com grupos de pacientes, seus familiares e a comunidade^(2)^. Nesse contexto, a atuação do enfermeiro apresenta um papel fundamental no desenvolvimento da enfermagem clínica, com utilização de protocolos de assistência, ações educativas que transcendem a construção conjunta de conhecimentos, orientação para o autocuidado, levantamento e planejamento de novas necessidades em saúde, o que proporciona inúmeras estratégias de cuidado^(8,9)^.

No cenário da APS, a visita domiciliar é a atividade principal do Agente Comunitário de Saúde (ACS), que está capacitado para realizar e atualizar o cadastro das famílias, auxiliar as equipes na identificação de áreas e situações de risco individual e coletivo, orientar sobre promoção e proteção da saúde, acompanhar o tratamento, mobilizar a comunidade para a conquista de ambientes favoráveis à saúde e notificar doenças que necessitam de vigilância^(10,11)^.

Além disso, os ACS desempenham um papel crucial no cuidado de pessoas com diabetes e hipertensão, tornando-se um elo essencial entre a comunidade e os serviços de saúde. Suas ações viabilizam o acompanhamento longitudinal dos pacientes com doenças crônicas e suas famílias, facilitando a criação de vínculos entre usuários e profissionais de saúde^(10)^.

Nesse cenário, a padronização das visitas domiciliares por meio de um instrumento específico é essencial para coletar dados precisos e consistentes; isso resulta em intervenções mais eficazes e personalizadas. Estudos mostram que a integração de tecnologias e ferramentas de suporte nas atividades dos ACS pode melhorar significativamente os desfechos clínicos de pessoas com diabetes e hipertensão, facilitando a identificação precoce de complicações e fatores de risco, e promovendo a prevenção de agravos^(11-13)^.

O uso de um instrumento validado fortalece ainda mais o vínculo entre a comunidade e os serviços de saúde, permitindo que os ACS forneçam orientações mais claras sobre hábitos saudáveis, adesão ao tratamento medicamentoso e monitoramento regular da glicemia e da pressão arterial^(11,12)^.

As tecnologias em saúde, portanto, constituem ferramentas importantes para o monitoramento de DM e HA. Assim, as informações podem ser fornecidas para apoiar o tratamento, o gerenciamento de doenças crônicas e a melhoria da capacidade e eficiência dos serviços de saúde^(12,13)^. Dessa maneira, esse instrumento pode contribuir significativamente para o processo de trabalho dos ACS e auxiliar os enfermeiros durante as consultas, obtendo dados que subsidiam o planejamento estratégico em saúde. Isso fortalece as orientações de cuidado, medidas de promoção à saúde e prevenção de complicações, visando à redução do impacto do DM e HA, por meio de diagnóstico precoce e cuidados adequados.

## OBJETIVOS

Descrever o processo de construção e evidência de validade de conteúdo de um instrumento para a visita domiciliar do Agente Comunitário de Saúde, no acompanhamento de pessoas com Diabetes *Mellitus* e Hipertensão Arterial, na Atenção Primária à Saúde.

## MÉTODOS

### Aspectos éticos

O protocolo de pesquisa, originado de uma tese de mestrado, foi apreciado e aprovado pelo Comitê de Ética em Pesquisa (CEP) da Universidade Federal de Santa Catarina (UFSC), cujo parecer está anexado à presente submissão. A coleta de dados obedeceu às orientações para procedimentos em pesquisas com qualquer etapa em ambiente virtual, publicadas pela Comissão Nacional de Ética em Pesquisa (CONEP). Para todos os participantes, foi enviado, por e-mail e aplicativo de mensagem, o Termo de Consentimento Livre e Esclarecido (TCLE), em formato PDF, devidamente assinado pelos pesquisadores. Ao “responder” o e-mail institucional do pesquisador contendo o TCLE e o questionário, ficou implícita a concordância com o TCLE e seu “aceite” em participar da pesquisa, dispensando-se a assinatura, conservando, contudo, a transparência e a rastreabilidade na relação entre participante da pesquisa e pesquisador.

### Desenho, local do estudo e período

Estudo metodológico, fundamentado nos preceitos preconizados a partir da proposta do modelo teórico do “Duplo Diamante” (*Double Diamond*)^(14)^, que descreve o desenvolvimento e validação de um instrumento composto pelas seguintes etapas: 1) Descobrir (*discover*); 2) Definir (*define*); 3) Desenvolver (*develop*); e 4) Entregar (*deliver*). Nesse sentido, este estudo foi realizado no período de setembro de 2022 a novembro de 2023, no âmbito de dez Unidades Básicas de Saúde de um município do Nordeste de Santa Catarina. Este estudo segue as diretrizes do *Revised Standards for Quality Improvement Reporting Excellence* (SQUIRE 2.0), que fornecem uma estrutura para relatar novos conhecimentos sobre como melhorar os cuidados de saúde.

### População ou amostra: critérios de inclusão e exclusão

O estudo contou com a participação de três amostras: agentes comunitários de saúde, enfermeiros e enfermeiros experts. Para a primeira e segunda etapas do estudo, foram convidados a participar os profissionais de saúde lotados na instituição, sendo a amostra selecionada segundo os critérios de inclusão: ser enfermeiro ou ACS atuante há, pelo menos, seis meses na ESF, em pleno domínio de consciência e que aceitasse participar do estudo, por meio da assinatura do TCLE. Foram excluídos os enfermeiros e ACS afastados por licença de saúde, maternidade ou férias.

No momento da coleta de dados, o município contava com 62 ACS e 16 enfermeiros atuantes em dez Unidades Básicas de Saúde, vinculadas a 12 Estratégias de Saúde da Família (ESF). Como três dessas unidades de saúde contavam com apenas um ACS cadastrado, almejou-se, nesta etapa, a participação de, no mínimo, um ACS de cada ESF, visando a identificar a realidade local de cada equipe. Além disso, para obter uma amostra representativa dos enfermeiros, objetivou-se a participação de, pelo menos, 50% dos enfermeiros atuantes.

Para a terceira etapa, na fase de evidência de validade de conteúdo, almejou-se o alcance de, no mínimo, sete *experts*, considerando o quantitativo ímpar para casos de empate nas avaliações. Os enfermeiros *experts* foram selecionados por meio de busca na Plataforma *Lattes* e, devido a dificuldades em receber retorno dos participantes e o número ser inferior ao mínimo preconizado, adotou-se, para dar seguimento à inclusão de novos *experts*, a busca pela técnica bola de neve (*snowball sampling* ^)(15)^.

Para a escolha dos *experts*, utilizou-se a pontuação adaptada do modelo Fehring^(16)^, em que os critérios de pontuação foram os seguintes: ser doutor ou mestre (3 pontos); possuir título de Especialista em Enfermagem em saúde pública, saúde coletiva ou saúde da família (3 pontos); especialização ou residência em saúde pública, saúde coletiva ou saúde da família (3 pontos); prática clínica atual mínima de dois anos em saúde pública, saúde coletiva, saúde da família ou assistência à pessoa com DM e HA (3 pontos); ter pesquisas ou artigos publicados sobre a temática do estudo (1 ponto); resumos publicados em saúde pública, saúde coletiva, saúde da família ou assistência à pessoa com DM e HA (1 ponto); participação em cursos ou congressos da área, com carga horária mínima de 4 horas (1 ponto). Foram incluídos os profissionais que, ao final da avaliação de critérios, obtiveram pontuação mínima de cinco pontos. Foram excluídos do estudo os profissionais que, mesmo selecionados, após duas tentativas de contato, não retornaram o *e-mail*.

### Protocolo do estudo

Na primeira etapa (*descobrir*), foi realizado um Diagnóstico Situacional (DS) com os ACS, por meio de um questionário semiestruturado, via *Google Forms*, com perguntas abertas e fechadas. O DS com os ACS teve por objetivo identificar a atuação frente ao atendimento, orientações de cuidados e acompanhamento das pessoas com DM e HA, sobretudo referente à visita domiciliar.

Na segunda etapa (*definir*), foi realizada uma revisão narrativa dos últimos cinco anos, nas seguintes fontes de dados: Biblioteca Virtual em Saúde (BVS), *Scientific Electronic Library Online* (SciELO), Portal Periódicos da CAPES, Medline via PubMed e normas técnicas, protocolos e diretrizes. Para tanto, utilizaram-se os seguintes descritores: DM, Hipertensão Arterial, APS, Tecnologia da Informação e Enfermagem.

Ainda, foi realizado um levantamento bibliográfico de busca não controlada em órgãos nacionais ligados à saúde da pessoa com HA e DM: Ministério da Saúde, Sociedade Brasileira de Cardiologia e Sociedade Brasileira de DM.

Concomitantemente, foi aplicado um questionário semiestruturado, com perguntas abertas e fechadas, para os enfermeiros atuantes nas unidades de saúde do município, a fim de levantar sugestões de conteúdos para compor o instrumento e identificar os problemas da prática e, a partir disso, elencar soluções que possam estar contidas na ferramenta. O questionário foi baseado na seguinte pergunta norteadora: “Quais informações a respeito das pessoas com diabetes e hipertensão são importantes estar contidas em um instrumento para auxiliar no acompanhamento dessas pessoas na APS, vinculado à visita domiciliar do ACS?”.

Para as duas primeiras etapas do estudo, o convite para participação na pesquisa incluía informações sobre os objetivos do estudo e o acesso ao TCLE. Além disso, foi enviado aos participantes um *link* de acesso ao questionário semiestruturado online, disponível no *Google Forms*.

A terceira etapa (*desenvolver*) foi realizada pensando em soluções para a construção do instrumento. Dessa maneira, a revisão da literatura, juntamente com a análise do diagnóstico situacional dos ACS e dos questionários dos enfermeiros, possibilitou a seleção dos conteúdos indispensáveis para a construção do instrumento. Assim, o protótipo do instrumento foi estruturado e organizado com o auxílio de ferramentas do *Microsoft Word*, com a descrição dos conteúdos para compor o instrumento.

Para garantir a qualidade e confiabilidade no conteúdo do produto, a versão preliminar do instrumento foi encaminhada para avaliação de conteúdo pelos experts da área, sendo que, para cada resposta da avaliação, foi empregado o cálculo do Índice de Validade de Conteúdo (IVC)^(17,18)^.

A procura por enfermeiros experts foi realizada por meio de uma busca na Plataforma Lattes, no portal do Conselho Nacional de Desenvolvimento Científico e Tecnológico (CNPq), com o auxílio da ferramenta “busca de currículo”, em “busca avançada”, utilizando os seguintes especificadores: DM, Hipertensão Arterial, APS, Enfermagem (busca por assunto), aplicando-se os filtros de formação acadêmica e atuação profissional, conforme os critérios de inclusão. Também foi utilizada a técnica de bola de neve, a fim de auxiliar no recrutamento, sendo realizada consulta ao universo relacional dos especialistas, os quais indicaram outros profissionais com os mesmos atributos.

Considerando a otimização do tempo de resposta, foi enviado um único *e-mail* com o convite para a participação na pesquisa, no qual foram explicadas a finalidade da participação, o *link* de acesso ao TCLE devidamente assinado pelos pesquisadores e o *link* de acesso ao formulário eletrônico para avaliação do instrumento, ambos construídos na plataforma *Google Forms*.

No formulário, foram utilizadas opções de resposta de múltipla escolha, definidas como de preenchimento obrigatório, com espaço para respostas abertas. Ao todo, compuseram o instrumento seis domínios, aos quais foi atribuída uma pontuação segundo a escala tipo Likert (1 = inadequado; 2 = parcialmente adequado; 3 = adequado; 4 = totalmente adequado) e um campo para considerações finais.

Após a devolutiva da avaliação pelos *experts* selecionados, foram realizadas as alterações com base nas propostas de mudanças e melhorias sugeridas pelos especialistas, sendo construída a versão final.

A quarta etapa (*entregar*) caracterizou-se pela entrega do instrumento para a Equipe de APS do município, para futura capacitação, informatização e implantação.

### Análise dos dados

As informações referentes aos dados qualitativos sobre o DS com os ACS e a análise dos questionários dos enfermeiros foram copiadas e agrupadas em um arquivo único, no programa *Word*® da *Microsoft Office*, versão *Windows* 10^®^. Os conteúdos encontrados na revisão da literatura e elencados pelos participantes foram agrupados por temas de afinidade e domínios. Estes foram selecionados até o momento em que houve saturação das informações, tanto na revisão da literatura quanto na análise dos questionários, e analisados de forma descritiva.

Para a análise dos dados qualitativos, foi utilizada a proposta de observação temática^(19)^. Para tanto, foram aplicados três momentos: a pré-análise, a exploração do material e o tratamento e a interpretação dos resultados obtidos. Quanto às informações sobre a caracterização dos participantes, estas foram organizadas em tabelas e planilhas, no programa *Excel* ^®^ da *Microsoft Office* ^®^, versão *Windows* 10^®^, e apresentadas a partir de dados relativos e absolutos, sendo analisadas de forma descritiva.

Os dados quantitativos, coletados de cada juiz participante, foram organizados em tabelas, em arquivo no programa *Microsoft Excel* ^®^, versão 2007, onde foram verificadas as pontuações atribuídas a cada um dos seis domínios avaliados, para, então, serem analisadas a partir de uma leitura reflexiva e estatística descritiva simples, com apresentação dos números absolutos e relativos.

No processo de análise da evidência de validade, calculou-se o IVC. Como critério de aceitação, foi estabelecida uma taxa de concordância aceitável de 80% (0,8) ou mais entre as avaliações dos juízes, para servir de critério de decisão sobre a pertinência e/ou aceitação do item, tanto para a avaliação de cada um deles quanto para a avaliação global do instrumento^(17)^. Os valores abaixo de 80% (0,8) seriam ajustados a partir das sugestões dos juízes e enviados novamente para avaliação, até atingir a confiabilidade desejada mínima de 80%. Porém, isso não foi necessário, já que, na primeira rodada, houve o alcance de consenso de 88 a 100% no IVC individual e 96% no IVC global.

## RESULTADOS

Participaram das primeiras e segundas etapas do estudo 41 profissionais, sendo 27 Agentes Comunitários de Saúde (ACS) e 14 enfermeiros. Em relação ao sexo, 95,1% dos participantes eram mulheres e 4,9% homens. Quanto à idade, 43,9% dos profissionais estavam na faixa etária de 40 a 50 anos. No que diz respeito à escolaridade dos ACS, 55,6% possuíam Ensino Médio completo. Entre os enfermeiros, 50% apresentavam título de especialização em Saúde da Família. Ao analisar o tempo de atuação dos profissionais na APS do município, constatou-se que 31,7% deles têm entre seis meses e dois anos de experiência, 26,8% atuam há mais de dez anos, 22,0% possuem entre dois e cinco anos de atuação, e 19,5% têm entre cinco e dez anos de experiência.

A revisão da literatura possibilitou a seleção dos conteúdos norteadores para a construção do instrumento, tais como: comorbidades; hábitos de vida (alimentação, atividade física, tabagismo e uso de drogas); situação de saúde atual (queixas clínicas relacionadas à HA e DM, monitoramento da pressão arterial e glicemia, cuidados com aplicação de insulina); e orientações de saúde sobre prevenção, controle e prevenção de complicações da HA e DM.

Com base nos dados obtidos no DS com os ACS e na análise do questionário com os enfermeiros, essas informações foram organizadas em cinco categorias temáticas, destacadas a seguir: 1) Dificuldades para visita domiciliar e acompanhamento do paciente; 2) Desafios na adesão ao tratamento; 3) Lacunas no cuidado – formação; 4) Lacunas no cuidado – ferramentas de apoio; e 5) Contribuições do ACS para o acompanhamento das pessoas com diabetes e hipertensão.

Dentre as dificuldades mencionadas, foi observado que há lacunas na formação dos profissionais, especialmente dos ACS, em relação ao manejo e às necessidades de cuidados para pessoas com diabetes e hipertensão, comprometendo a qualidade e a eficácia do atendimento. Dessa forma, ao evidenciar que a equipe necessita de ferramentas tecnológicas de suporte para auxiliar no acompanhamento dos pacientes e nas orientações de cuidados, foi possível realizar a construção e estruturação do instrumento para coleta, registro dos dados e evidência de validade do conteúdo pelos enfermeiros *experts*.

Para a etapa de evidência de validade do conteúdo, participaram nove enfermeiros. Em relação ao perfil profissional dos juízes que participaram desse processo, todas eram do sexo feminino (100%), sendo que a maioria se encontrava na faixa etária entre 20 e 50 anos (66,6%). Do total, 33,3% eram doutoras em Enfermagem, 88,8% mestras em Enfermagem e 100% possuíam título de especialista. A maior parte delas (88,8%) possuía mais de dez anos de atuação como enfermeira. Quanto à instituição de vínculo empregatício atual, 55,6% atuavam em Secretarias Municipais de Saúde e 44,4% em instituições de ensino superior.

O instrumento utilizado para a evidência de validade do conteúdo, encaminhado aos enfermeiros *experts*, foi composto por seis domínios, subdivididos nos seguintes aspectos:

### Domínio 1 – Diagnóstico Clínico:

DM; Hipertensão Arterial.

### Domínio 2 – Dados de Identificação:

Nome, nome social, nome da mãe, nome do pai, data de nascimento, sexo, estado civil, etnia, cartão do Sistema Único de Saúde (SUS), endereço, escolaridade, ocupação, telefone e responsável familiar.

### Domínio 3 – Comorbidades e Condição de Saúde Referida:

DM tipo 1 e tempo de diagnóstico; DM tipo 2 e tempo de diagnóstico; não sabe informar o tipo e tempo de diagnóstico; HÁ e tempo de diagnóstico; acidente vascular cerebral (derrame cerebral); colesterol alto; infarto agudo do miocárdio; histórico familiar de diabetes; peso referido (kg); altura referida (cm).

### Domínio 4 – Hábitos de Vida:

Tabagismo; uso de drogas; consumo de bebidas alcoólicas; prática de atividade física; alimentação; ingestão de água.

### Domínio 5 – Situação de Saúde Atual:

**5.1 – Hipertensão Arterial:** Possui aparelho para aferição da Pressão Arterial (PA) em casa; valor da última PA referida; situações de queixa de sintomas de PA alta, relatadas pelo paciente.

**5.2 – DM:** Frequência da realização do teste de glicemia capilar; situações de hipoglicemia e hiperglicemia.

**5.3 – Paciente com diagnóstico de DM em uso de insulina:** Uso de caneta ou seringa para insulina; local de armazenamento da insulina aberta e fechada; rodízio dos locais de aplicação da insulina; responsável pela aplicação da insulina; descarte dos resíduos dos materiais perfurocortantes; dificuldades com os cuidados relacionados ao uso da insulina.

**5.4 – Investigação de Saúde Atual:** Data do último exame de sangue; responsável por retirar o medicamento na farmácia da unidade; local de armazenamento dos medicamentos; receita de medicamento de uso contínuo dentro da validade; presença de feridas (lesões de pele) nos membros inferiores; histórico de hospitalizações relacionadas ao DM ou à HA nos últimos 30 dias; data da última consulta na unidade de saúde; situação vacinal e autocuidado.

### Domínio 6 – Orientações de Saúde:

Inclui as orientações a serem repassadas à comunidade: esclarecer sobre os fatores de risco para as doenças cardiovasculares, orientando quanto às medidas de prevenção, com ênfase na evitação de hábitos prejudiciais, como o tabagismo e o consumo excessivo de álcool; reforçar o uso correto dos medicamentos, conforme prescrição médica; auxiliar o paciente a seguir as orientações recebidas pela equipe de saúde sobre a adesão a uma dieta saudável, rica em fibras, pobre em açúcares e gorduras, com baixo teor de sal e ingestão adequada de água, considerando a realidade e as necessidades de cada paciente; apoiar o paciente na adoção de prática regular de atividade física apropriada à sua condição de saúde; reforçar as orientações da equipe de saúde sobre o descarte dos resíduos dos pacientes em uso de insulinoterapia, que deve ser realizado em recipiente rígido e resistente, e, quando cheio, entregue na unidade básica de saúde para o descarte adequado; orientar e encaminhar o paciente para o agendamento de consulta na unidade, em caso de queixas clínicas; realizar orientações sobre a vacinação, conforme preconizado pelo Programa Nacional de Imunizações, e encaminhar para a unidade de saúde os pacientes com vacinas em atraso; orientar sobre os horários de funcionamento da unidade, da sala de vacinação, de consultas e exames, bem como sobre a renovação de receitas; reforçar a necessidade de o paciente comparecer às consultas e realizar os exames solicitados pelas equipes de saúde; incentivar a participação nos grupos de educação em saúde da unidade.

As principais modificações sugeridas pelos juízes foram: substituir “estado civil” por “situação conjugal”; acrescentar a investigação da hipertensão gestacional; incluir se o paciente possui glicosímetro em casa e se este é próprio ou da unidade de saúde; acrescentar quais são os horários da realização do teste de glicemia capilar; investigar os horários mais frequentes em que ocorrem episódios de hipoglicemia; excluir a investigação do esquema vacinal.

Todas essas modificações foram incorporadas ao instrumento, incluindo as observações do juiz (J2), que classificou o Domínio 2 como parcialmente adequado e recomendou a substituição de “estado civil” por “situação conjugal” (Quadro 1). A única recomendação que não foi acatada foi a exclusão do item de investigação do esquema vacinal, pertencente ao Domínio 6 sobre as Orientações de Saúde, pois essa sugestão foi feita por apenas um juiz (J9).

**Quadro 1:** Itens que compuseram o instrumento final e os cálculos de IVC, após a avaliação de conteúdo dos *experts*, Florianópolis, Santa Catarina, Brasil, 2023

ITEM/ DOMÍNIO	J1	J2	J3	J4	J5	J6	J7	J8	J9	IVC INDIVIDUAL
**1**	4	3	4	4	3	3	4	3	3	**1,00**
**2**	4	2	3	4	3	3	4	4	3	**0,88**
**3**	4	3	4	4	3	3	4	4	3	**1,00**
**4**	4	4	3	4	3	3	3	4	3	**1,00**
**5**	4	3	4	4	3	3	3	3	3	**1,00**
**6**	4	3	3	4	3	3	4	4	1	**0,88**
**IVC TOTAL**										**0,96**

*J – Juiz; IVC – Índice de Validação de Conteúdo.*

Assim, foi possível desenvolver a versão final do instrumento após a validação dos conteúdos realizada por enfermeiros *experts*. O instrumento foi disponibilizado à equipe da APS do respectivo serviço para implementação no módulo de visita domiciliar dos ACS. A aplicabilidade desse instrumento ocorrerá durante as visitas domiciliares e, por meio do registro das informações inseridas nos tablets, os ACS poderão aplicá-lo e atualizar os dados periodicamente, atendendo às necessidades da equipe.

## DISCUSSÃO

O presente estudo destaca a importância do desenvolvimento e da evidência de validade de conteúdo de ferramentas tecnológicas que auxiliem os ACS no acompanhamento de pessoas com doenças crônicas como hipertensão e diabetes. Experiências anteriores, como a validação de aplicativos para o cuidado dos pés de pessoas vivendo com diabetes, demonstraram que tais tecnologias são não só viáveis, mas também confiáveis para aplicação prática, fomentando o autocuidado e prevenindo complicações como hospitalizações e amputações^(20)^.

A pesquisa revelou a importância de que as tecnologias desenvolvidas na área da saúde atendam efetivamente às demandas identificadas e sejam relevantes para o contexto específico em que serão aplicadas. Isso requer que o pesquisador tenha uma compreensão profunda das necessidades e características do público-alvo^(21)^. Embora essa questão não tenha sido o foco deste estudo, é importante ressaltar que a relevância das informações coletadas foi reforçada pela participação ativa dos ACS e enfermeiros na elaboração do instrumento, profissionais que estão diretamente envolvidos no cuidado aos usuários. Além disso, a incorporação de sugestões de especialistas também contribuiu para garantir que as tecnologias atendam às necessidades reais do contexto.

Durante a evidência de validade do instrumento, os especialistas recomendaram a inclusão do histórico de hipertensão gestacional. As síndromes hipertensivas durante a gravidez são indicadores significativos de risco futuro, pois pacientes que desenvolvem qualquer tipo de HA durante a gestação, como a pré-eclâmpsia precoce, têm maior probabilidade de enfrentar desfechos adversos, como prematuridade. Essas condições elevam a chance de desenvolver hipertensão crônica em até três a quatro vezes^(2)^.

Além disso, os juízes sugeriram incluir no estudo a investigação sobre se as pessoas com diabetes utilizam glicosímetro próprio ou fornecido pela unidade de saúde, além de considerar os horários de realização do teste de glicemia capilar e os momentos mais frequentes de hipoglicemia. Em uma pesquisa realizada no Hospital Universitário do Distrito Federal sobre o acesso à insulinoterapia para pessoas com diabetes, observou-se que a maioria dos participantes obtinha seus medicamentos, incluindo insulina (93,3%) e antidiabéticos orais (50,0%), no serviço público, especialmente nas UBS^(22)^.

Quanto aos insumos para monitoramento da glicemia capilar, como glicosímetro, fitas e lancetas, também foram adquiridos predominantemente no setor público (93,3%). É importante mencionar que o fornecimento do glicosímetro pelo SUS está regulamentado por portaria específica^(22)^. A investigação sugerida pelos juízes, de verificar se os pacientes utilizam glicosímetro próprio ou da unidade de saúde, é relevante para orientar os ACS no cuidado às pessoas com diabetes. Esses dados destacam a necessidade de ferramentas eficazes, como a tecnologia educacional sobre insulinoterapia, para atender às necessidades específicas das pessoas com diabetes na APS, garantindo cuidado personalizado e prevenção de complicações.

Em relação ao controle glicêmico, uma pesquisa investigou a correlação entre a consulta de enfermagem e o cumprimento de ações de autocuidado e práticas seguras em insulinoterapia por pessoas com diabetes. Foi observado que 41,9% dos participantes relataram episódios de hipoglicemia e 76,7% apresentaram hiperglicemias constantes no Automonitoramento Glicêmico (AMG), por meio da aferição da glicemia capilar. Quanto à aplicação de insulina, 87,4% dos pacientes realizavam autoaplicação, enquanto 12,6% tinham a aplicação realizada por familiares ou cuidadores^(23)^. Esses resultados ressaltam a importância do acompanhamento contínuo e da educação em saúde para melhorar o manejo da doença e a qualidade de vida das pessoas vivendo com diabetes na APS.

Em relação ao item sobre investigação de vacinas em atraso, pertencente ao domínio “Orientações de Saúde”, foi sugerida sua retirada por apenas um juiz. Contudo, optou-se por manter o item. A importância desse acompanhamento é destacada pelo fato de que pessoas com diabetes e hipertensão são consideradas grupos de risco para diversas doenças preveníveis. Um estudo realizado nos Estados Unidos, que analisou pacientes com COVID-19 hospitalizados, revelou que as comorbidades mais prevalentes foram hipertensão (56,6%), obesidade (41,7%) e diabetes (33,8%)^(24)^.

Nesse contexto, as tecnologias em saúde validadas desempenham um papel importante ao contribuírem para um tratamento efetivo na prevenção de complicações e agravos^(25,26)^. Além disso, as tecnologias estão cada vez mais presentes no cotidiano das pessoas e têm se expandido para o ambiente de trabalho, sendo essenciais para o desenvolvimento de novos recursos e estratégias educativas. Assim, essas ferramentas tecnológicas são aliadas fundamentais em políticas públicas voltadas para o combate à diabetes e à hipertensão, além de ajudarem a preencher lacunas de conhecimento existentes sobre essas condições.

### Limitações do estudo

Como limitação deste estudo, aponta-se a dificuldade no recebimento das devoluções dos instrumentos de avaliação por parte dos juízes. Destaca-se, ainda, a necessidade de pesquisas que possam avaliar a efetividade da implantação do instrumento, seja na capacitação dos ACS, na análise dos indicadores de saúde ou na melhora da adesão terapêutica e, consequentemente, na prevenção de riscos e agravos.

### Contribuições para a área da enfermagem, saúde ou política pública

O desenvolvimento de tecnologias na APS desempenha um papel determinante no avanço das políticas públicas voltadas à prevenção de doenças crônicas. A construção de instrumentos alinhados às necessidades reais dos profissionais de saúde que atuam na rede de APS contribui, a médio prazo, para a promoção de cuidados individualizados. A longo prazo, espera-se que essas iniciativas reduzam significativamente os riscos de complicações relacionadas às doenças crônicas, melhorando assim a qualidade de vida dos pacientes. Além disso, o modelo pode ser adaptado e implementado em diversas realidades dentro da APS, ampliando seu impacto e eficácia. A coleta de dados pelos ACS proporciona uma visão detalhada do perfil de saúde local, permitindo que a equipe de saúde, especialmente a enfermagem, planeje intervenções estratégicas e desenvolva cuidados mais adequados às necessidades da comunidade.

## CONCLUSÕES

O instrumento proposto demonstrou índices satisfatórios de validade de conteúdo, destacando-se como uma ferramenta capaz de padronizar e orientar o trabalho dos ACS durante as visitas domiciliares às pessoas com diabetes e hipertensão na APS. Ao validar o conteúdo do instrumento de coleta de dados, este estudo representa um passo significativo para aprimorar o cuidado oferecido na APS, fornecendo aos profissionais ferramentas eficazes para acompanharem, de forma mais precisa e integrada, as pessoas com diabetes e hipertensão. Essa abordagem integrada é essencial para promover a saúde, prevenir complicações e, consequentemente, melhorar a qualidade de vida das pessoas que vivem com diabetes e hipertensão. Além disso, a utilização deste instrumento pode contribuir para a redução de custos e dos impactos que o DM e a HA geram aos serviços de saúde.

## Data Availability

Os dados de pesquisa estão disponíveis em repositório: https://doi.org/10.48331/scielodata.X6EGTK.
